# A penta­nuclear iridium(III) hydride cluster: aggregation of an iridium(I) precatalyst

**DOI:** 10.1107/S2056989025008023

**Published:** 2025-09-16

**Authors:** Ben. J. Tickner, Richard J. Gammons, Carlos Platas-Iglesias, Adrian C. Whitwood, Simon B. Duckett

**Affiliations:** a Centre for Hyperpolarisation in Magnetic Resonance, University of York, Heslington, YO10 5NY, United Kingdom; b Department of Chemistry, University of York, Heslington, YO10 5DD, United Kingdom; c Centro Interdisciplinar de Química e Bioloxía (CICA), Facultade de Ciencias, Universidade da Coruña, A Coruña, 15001, Spain; Vienna University of Technology, Austria

**Keywords:** crystal structure, Ir clusters, polynuclear cluster, hydrides, catalysis, bridging hydride ligands., crystal structure

## Abstract

The crystal structure of a unique penta­nuclear Ir cluster, tetra­kis­[1,3-bis­(2,4,6-tri­methyl­phen­yl)-1,3-di­hydro-2*H*-imidazol-2-yl­idene-κ*C*^2^]carbonyldi-μ_3_-hydrido-tetra-μ_2_-hydrido-nona­hydridopenta­iridium(III), [Ir_5_(μ_3_-H)_2_(μ_2_-H)_4_H_9_(C_21_H_24_N_2_)_4_(CO)], has been refined from X-ray data and supported by density functional theory (DFT) calculations. The five iridium(III) sites of the cluster form a trigonal–bipyramidal structure: three are located in the equatorial triangular plane and are capped by axial metal sites above and below the centre of this plane. Four of these iridium atoms are associated with an N-heterocyclic carbene ligand, and a fifth, which is located in the equatorial plane, is bonded to a CO ligand, which must come from the methanol solvent. The 15 hydride ligands in the cluster could not be located in electron-density difference maps and their locations were optimized by using DFT approaches to calculate the lowest energy structure. These methods revealed the presence of nine terminal, four μ_2_-, and two μ_3_-bridging hydrides, which unusually cap faces of three metal atoms. The cluster formed from reaction of an Ir^I^ precursor with H_2_ and NaOMe base in methanol, and it likely reflects an example of a catalytic deactivation product when active Ir^III^ hydrogenation, isotope exchange, or signal amplification by reversible exchange (SABRE) catalysts aggregate in solution to form crystalline or other solid-state products.

## Abstract 2

The single-crystal X-ray structure analysis of a penta­nuclear iridium hydride cluster containing four N-heterocyclic carbenes and a CO ligand was supported by DFT-calculations. The penta­nuclear iridium core exhibits a trigonal–bipyramidal structure and the 15 hydride sites show terminal, μ_2_- and μ_3_-bridging coordination modes.

## 1. Chemical context

Polynuclear clusters can feature as key inter­mediates or deactivation products in metal-catalysed reactions. Notably, metallic clusters can be highly useful as they may display properties somewhere between single-site homogeneous systems and higher order nanoparticles (Tang & Zhao, 2020 ▸). Accordingly, their preparation and structural elucidation can further understanding of the role such species play in catalysis. In this work we describe a metal hydride cluster containing five iridium(III) atoms. This species is formed from the 16-electron iridium(I) precursor [IrCl(COD)(IMes)] (where COD is cis,cis-1,5-cyclo­octa­diene and IMes is 1,3-bis­(2,4,6-trimethyl-phen­yl)imidazol-2-yl­idene), which is commonly used as a precatalyst for hydrogenation (Tickner *et al.*, 2019 ▸), hydrogen isotope exchange (Cochrane *et al.*, 2013 ▸; Timofeeva *et al.*, 2020 ▸; Kerr *et al.*, 2021 ▸), and the signal amplification by reversible exchange (SABRE) hyperpolarization method (Cowley *et al.*, 2011 ▸). In these cases, active catalysts are usually based on mononuclear Ir sites, although reactions of [IrCl(COD)(IMes)] with H_2_ and various ligands can lead to dimeric Ir byproducts (Tickner & Zhivonitko, 2022 ▸). In some processes, such as hydrogen isotope exchange, dimeric Ir species have been indicated to exhibit catalytic activity and play a role in the overall catalysis (Tickner *et al.*, 2025 ▸). However, in many examples utilising this Ir^I^ precursor, aggregation of the resultant {Ir^III^H_2_} units can lead to a decrease in general catalytic efficiency over time, particularly in cases where [IrCl(COD)(IMes)] is used as a SABRE catalyst (Tickner & Zhivonitko, 2022 ▸). In fact, [IrCl(COD)(IMes)] has been reported to aggregate into trinuclear and tetra­nuclear species upon its reaction with NaOMe and H_2_ in methanol (Tickner *et al.*, 2024 ▸), and these catalytically inactive products likely form during routine SABRE catalysis as low concentration byproducts. To this end, we were able to extend these observations further by the preparation and growth of single crystals of a higher order penta­nuclear Ir cluster, which were examined using X-ray diffraction studies and were formed from the reaction of an Ir^I^ precatalyst with a base in methanol.
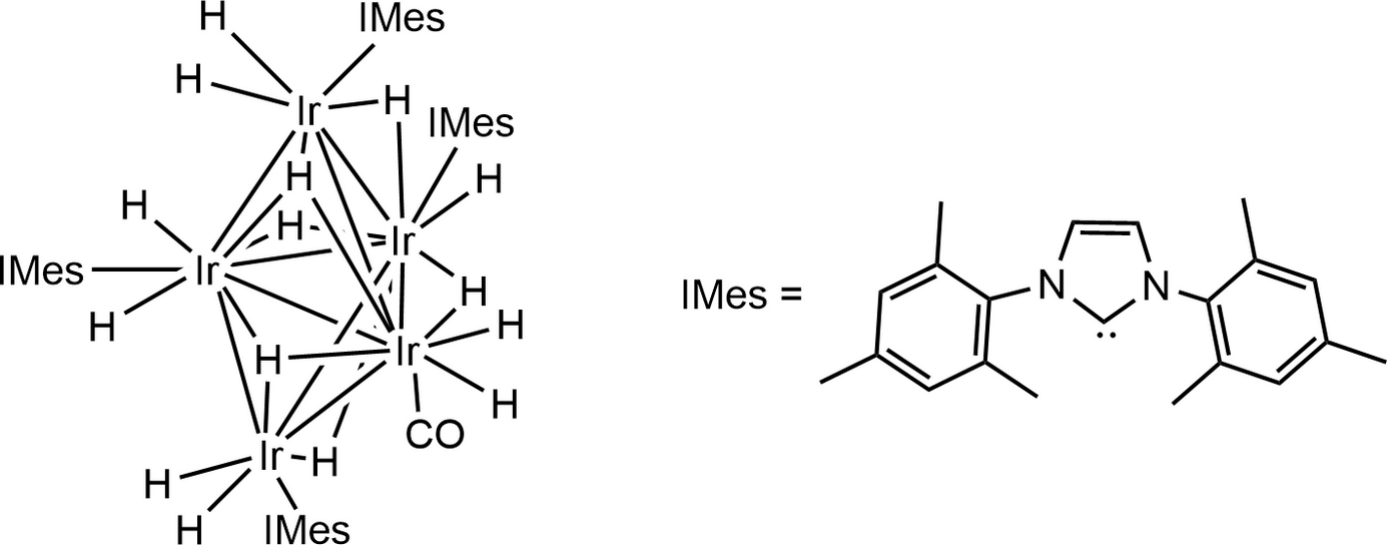


## 2. Structural commentary

**Figure 1:**
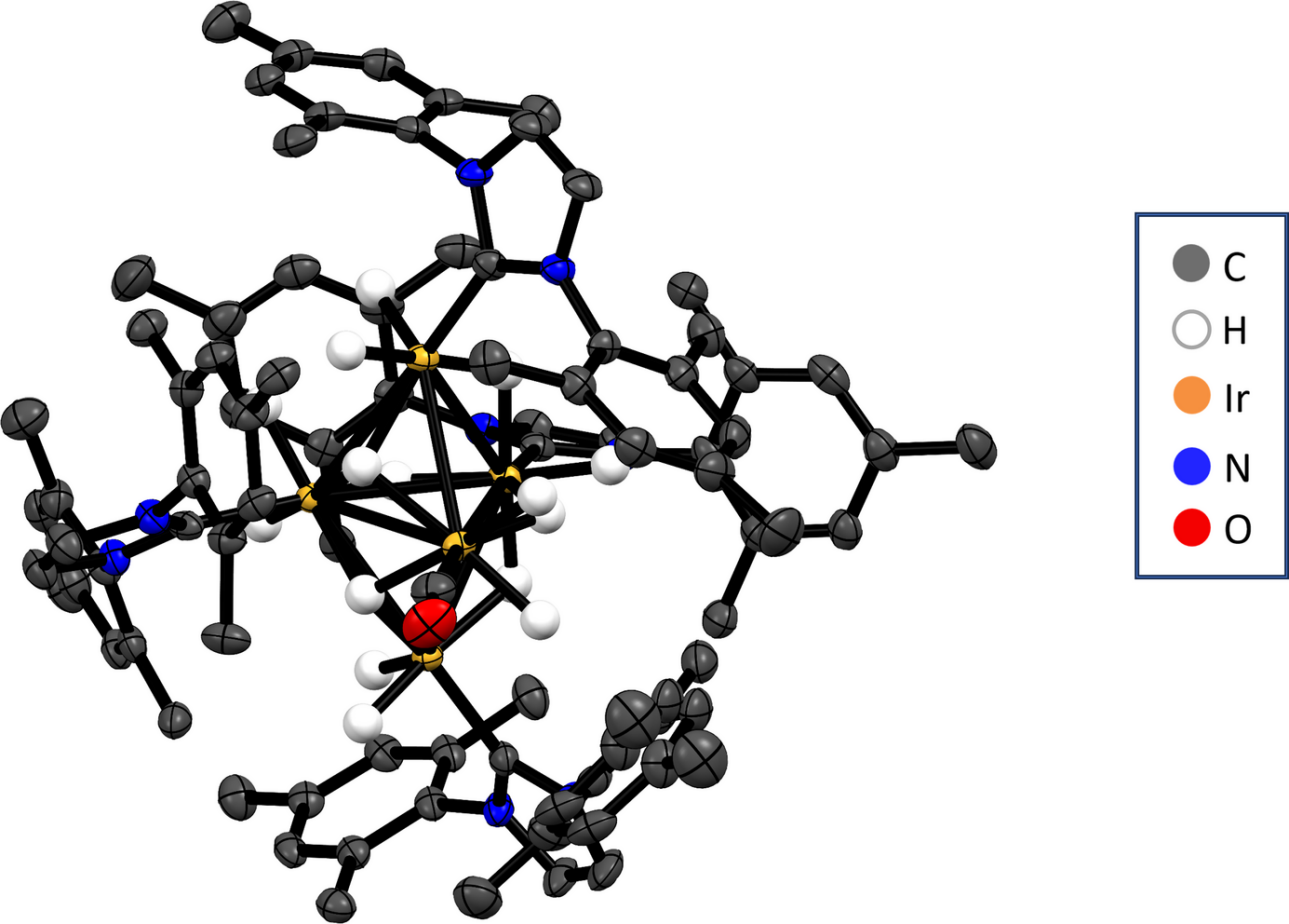
The mol­ecular structure of [Ir_5_(μ_3_-H)_2_(μ_2_-H)_4_(H)_9_(CO)(IMes)_4_], with displacement ellipsoids given at the 50% probability level. Note that non-hydride hydrogen atoms are omitted for clarity.

**Figure 2:**
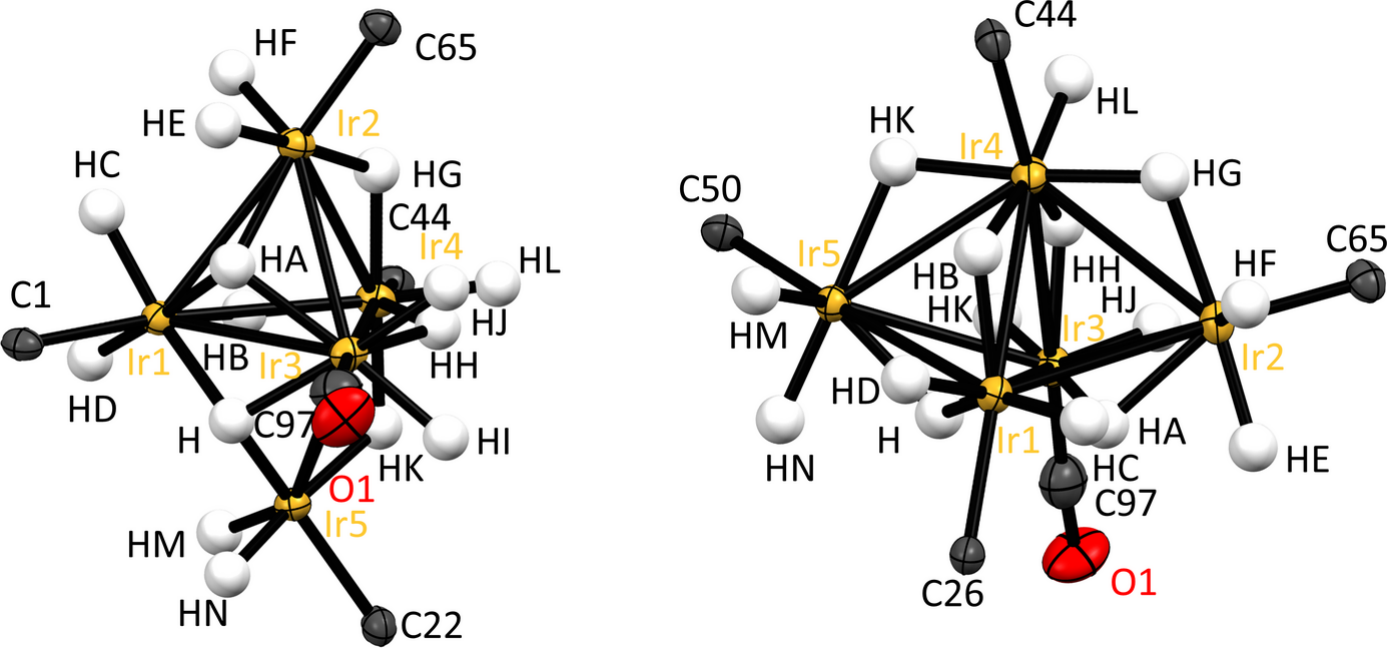
Penta­nuclear core of [Ir_5_(μ_3_-H)_2_(μ_2_-H)_4_(H)_9_(CO)(IMes)_4_], with displacement ellipsoids given at the 50% probability level. Note that only the carbene carbon atoms of the NHC ligands are shown.

The mol­ecular structure of the title compound, [Ir_5_(μ_3_-H)_2_(μ_2_-H)_4_(H)_9_(CO)(C_21_H_24_N_2_)_4_] where C_21_H_24_N_2_ is the N-heterocyclic carbene ligand IMes, is displayed in Fig. 1 ▸. The metallic core adopts a trigonal–bipyramidal shape consisting of three metal sites in the equatorial plane (Ir1, Ir3, Ir4), capped with iridium sites above and below the equatorial plane (Ir2, Ir5) (Fig. 2 ▸). Four of these five Ir atoms are ligated by the N-heterocyclic carbene IMes (Ir1, Ir2, Ir4, Ir5). However, one iridium site (Ir3) is bound to a CO ligand and its location in the equatorial plane is likely related to steric constraints associated with fitting three {Ir(IMes)} units in the same equatorial plane. Placing a sterically costly {Ir(IMes)} unit in the axial sites is likely favoured compared to the equatorial sites, as when in the axial position these ligands largely point away from the rest of the cluster. The presence of a CO ligand within this cluster is unanti­cipated given that it has not been prepared in the presence of carbon monoxide. The source of this ligand is likely solvent methanol, which can decompose to form CO and di­hydrogen. It is noteworthy that we are not aware of any reports of single site IrI or Ir^III^-catalysed methanol decomposition to CO, and this reaction has not been observed in many other examples where dimeric (or even up to tetra­meric) Ir clusters are present as byproducts (Tickner *et al.*, 2024 ▸; Tickner & Zhivonitko, 2022 ▸). However, it has been reported to occur on heterogeneous iridium surfaces (Wang *et al.*, 2013 ▸; Weststrate *et al.*, 2007 ▸). The presence of CO in the cluster reported here suggests the cluster could have novel catalytic properties inter­mediate between single-site catalysts and the larger solid supported systems that are typically used for methanol decomposition (Chen *et al.*, 2019 ▸; Matsumura *et al.*, 1998 ▸, 2000 ▸; Ranaweera *et al.*, 2017 ▸; Shen & Matsumura, 2000 ▸).

The axial Ir sites (Ir2, Ir5), and two within the trigonal plane (Ir1, Ir3) are each associated with two terminal hydride ligands. The two NHC-bound Ir sites within this equatorial plane (Ir1, Ir4) are distinct as one only (Ir4) contains one terminal hydride. Accordingly, the whole cluster has a pseudo-mirror plane running through the three equatorial Ir sites, but no perpendicular symmetry planes due to the different arrangement of the hydride ligands on effectively inequivalent Ir sites. The four μ_2_ hydrides bridge adjacent Ir–Ir pairs, two of which are between Ir sites within the equatorial plane and the remaining two link equatorial and axial Ir sites. Two of the 15 hydrides in the cluster cap three Ir sites, with these two μ_3_-bridging hydrides both capping the same two equatorial Ir atoms but different axial sites.

The bond lengths between the atoms within the penta­nuclear core, and the ligands directly bound to them, are shown in Table 1 ▸. The nine Ir—Ir distances range from 2.8438 (3) to 3.0067 (3) Å. As the atomic radius of iridium is 1.36 Å (Van Zon *et al.*, 1993 ▸; Kirschen *et al.*, 1995 ▸) these distances are comparable to the sum of the atomic radii of adjacent Ir sites. Therefore, metallophilic inter­actions are likely to play a large role in the bonding within the cluster (Sculfort & Braunstein, 2011 ▸). The Ir—CO bond length is shorter [1.752 (6) Å] compared to the Ir—C(IMes) bond lengths [>1.950 (5) Å] and is likely related to electronic and steric factors. Of the metal–hydride inter­actions, terminal Ir—H bonds are generally shorter whereas hydrides spanning more than one metal tend to have longer metal–hydride distances.

## 3. Supra­molecular features

**Figure 3:**
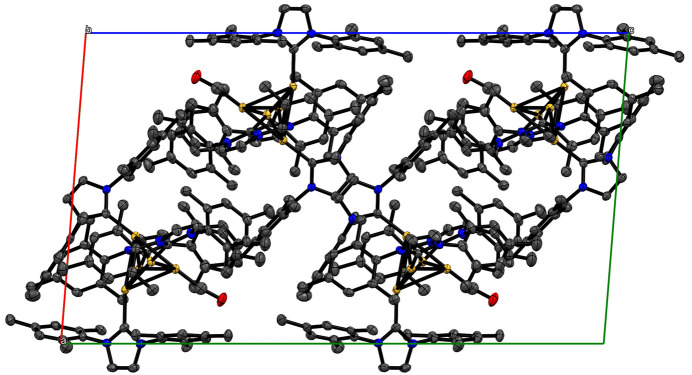
Crystal packing of [Ir_5_(μ_3_-H)_2_(μ_2_-H)_4_(H)_9_(CO)(IMes)_4_], shown along the crystallographic *b* axis. Displacement ellipsoids are given at the 50% probability level and hydrogen atoms are omitted for clarity.

The crystal does not contain any solvent-filled voids, or solvent of crystallization. Long-range inter­actions between the mol­ecules of [Ir_5_(μ_3_-H)_2_(μ_2_-H)_4_(H)_9_(CO)(IMes)_4_] involve inter­actions between the IMes ligands on adjacent mol­ecules. The shortest of these is a 2.294 Å inter­action between two hydrogen atoms on terminal mesityl methyl groups between different IMes ligands (H30*C* and H61*B*). A similar 2.273 Å inter­action exists between an imidazole CH hydrogen atom within the IMes ligand and a hydrogen atom on the meta CH_3_ group of the mesityl ring of a different IMes (H67 and H34*A*). Inter­actions between the IMes ligands on different mol­ecules play a role in the crystal packing, but there do not seem to be many π-stacking inter­actions. Instead, the terminal methyl groups of IMes ligands sit above the plane of a mesityl ring on another IMes ligand, rather than the two rings being in parallel planes. This is evidenced by an almost perpendicular 88.09° angle between the C25, C26, C28, C29, C32, C33 mesityl plane on one IMes and the C56, C57, C59, C60, C62, C63 mesityl plane on an adjacent IMes and a short 2.357 (5) Å distance between a terminal methyl H atom on IMes (H30C) and the C56, C57, C59, C60, C62, C63 mesityl plane. The crystal packing is shown in Fig. 3 ▸.

## 4. Database survey

A search of the Cambridge Structure Database (CSD, Version 5.45, update November 2023; Groom *et al.*, 2016 ▸) revealed crystal structures for a range of other iridium clusters, several of which contain a higher number of iridium sites, i.e. greater than the five observed in the cluster reported here (Adams *et al.*, 2005 ▸; Della Pergola *et al.*, 1990 ▸, 1998 ▸; Pierpont *et al.*, 1978 ▸; Pergola *et al.*, 1999 ▸). However, these typically contain CO and/or phosphine ligands with CO often acting as a bridging ligand. Iridium hydride clusters are rarer and examples of them typically involve fewer Ir sites, four or fewer (Xu *et al.*, 2009 ▸; Tickner *et al.*, 2024 ▸; Tang *et al.*, 2011 ▸). The crystal presented here reflects an inter­esting example bridging these extremes as it is predominantly an iridium hydride cluster, with a high number of metal atoms. The majority of these metal–hydride clusters contain terminal, or μ_2_-bridging hydrides. The cluster reported herein provides an unusual example containing two μ_3_-bridging hydrides. Structures containing hydrides spanning three metal atoms have been reported before, but examples are rare (Ferrer *et al.*, 1992 ▸; Andrews *et al.*, 1980 ▸).

**Figure 4:**
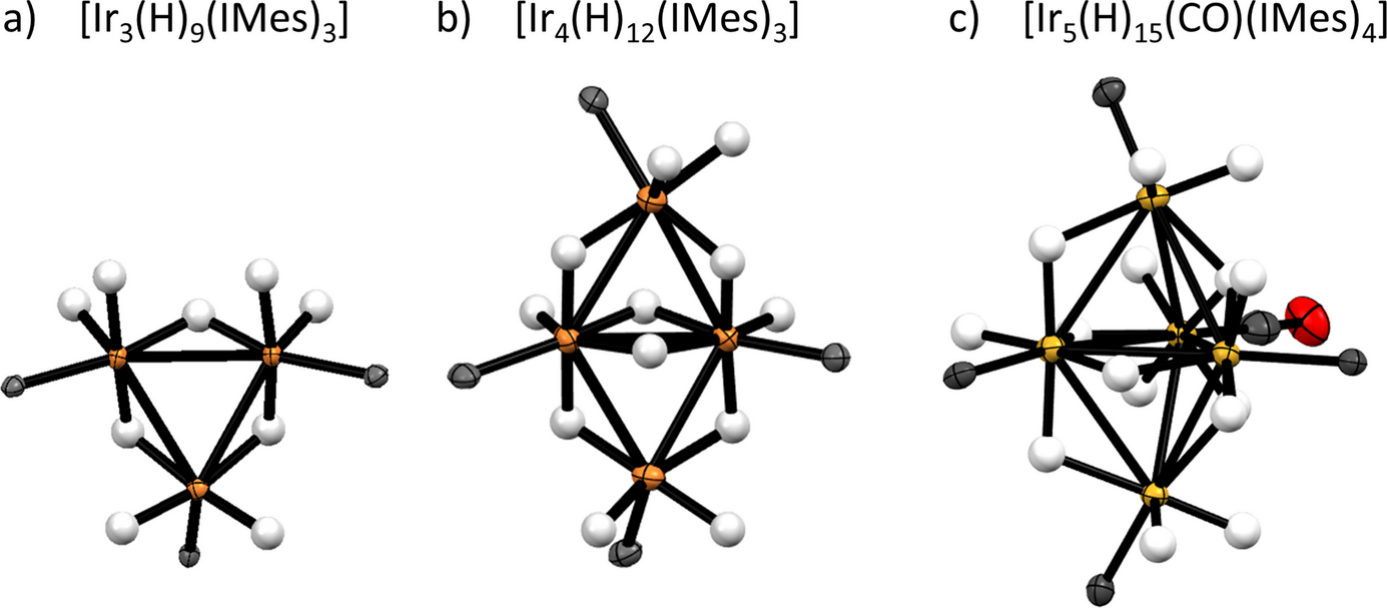
Similarity of [Ir_5_(H)_15_(CO)(IMes)_4_] (shown in c) to other reported (*a*) Ir_3_ and (*b*) Ir_4_ clusters (Tickner *et al.*, 2024 ▸). All of these closely related clusters are formed from reaction of the Ir(I) precursor [IrCl(COD)(IMes)] with NaOMe and H_2_ in methanol. Displacement ellipsoids are given at the 50% probability level and only the carbene carbon atoms of the NHC ligands are shown.

An analysis of 35 Ir—Ir bond lengths for similar Ir^III^–IMes hydride dimers, trimers, and tetra­mers revealed an average Ir—Ir distance of 2.77 ± 0.16 Å (mean ± standard deviation), which is comparable to the average Ir—Ir distances in the penta­nuclear cluster described here (2.94 ± 0.05 Å). The average Ir—IMes bond length in the title compound is 2.00 ± 0.05 Å and is consistent with other Ir^III^—IMes bond lengths in related crystal structures (2.02 ± 0.05 Å, *n* = 61). The shape of the Ir_5_ core in [Ir_5_(H)_15_(CO)(IMes)_4_] consists of an equatorial Ir_3_ plane, with axial Ir sites above and below this plane. It is closely related to that of a similar [Ir_3_(H)_9_(IMes)_3_] cluster in which three core Ir atoms are in a trigonal–planar shape. The related tetra­meric butterfly cluster [Ir_4_(H)_12_(IMes)_4_] has also been reported consisting of two fused trigonal Ir_3_ units along a shared Ir—Ir axis (Fig. 4 ▸). Both these trimeric and tetra­meric Ir clusters have been reported to form from the same reaction of [IrCl(COD)(IMes)] with NaOMe in methanol as used here (Tickner *et al.*, 2024 ▸). These clusters could be important precursors to the formation of higher order Ir aggregates, such as nanoparticles. Formation of these deactivation products is likely linked to a drop in catalytic performance as a function of reaction time when Ir^I^ precursors are used for hydrogenation or SABRE (Tickner *et al.*, 2019 ▸, 2020 ▸).

## 5. Synthesis and crystallization

The penta­nuclear Ir cluster was obtained by reaction of [Ir(Cl)(COD)(IMes)] (2.00 mg) with a solution of NaOMe (7.2 µl of a 25% *w*/*w* solution of NaOMe in methanol) and H_2_ (3 bar) in methanol-*d*_4_ (0.6 ml) for several days at room temperature (*ca* 291 K) in a 5 mm NMR tube with a J. Youngs tap. All reagents were added to the NMR tube before it was degassed using three freeze–pump–thaw cycles on a high vacuum line. Hydrogen gas was then added by connecting a hydrogen cylinder with a regulator to the high vacuum line and opening the lid of the NMR tube. The tube was vigorously shaken to dissolve the hydrogen gas. After reaction for several days at room temperature, the solution was cooled to 278 K in a fridge for several weeks to form single crystals, which were found by X-ray diffraction to be the title compound. Under these conditions, we found crystallization of the title compound to be extremely challenging, and crystals of the title compound formed in a small percentage of samples prepared in this way. Note that the crystals prepared as described can be [Ir_3_(H)_9_(IMes)_3_] or [Ir_4_(H)_12_(IMes)_4_], with crystallization of the former more likely. More details about these other products, their formation, and this reaction, have been reported elsewhere (Tickner *et al.*, 2024 ▸).

## 6. Refinement and accompanying DFT calculations

**Figure 5:**
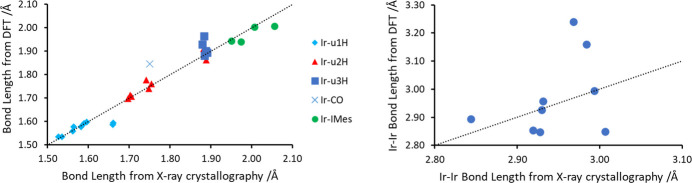
Comparison of bond lengths for [Ir_5_(H)_15_(CO)(IMes)_4_] determined from X-ray crystallography and density functional theory.

Crystal data, data collection and structure refinement details are summarized in Table 2 ▸. All iridium atoms were confirmed to be in a +III oxidation state from the Ir—IMes bond lengths (see section 4), which confirmed that there must be 15 hydride ligands. These could not be located by electron-density difference maps and, therefore, density functional theory (DFT) calculations were performed to obtain the lowest energy shape of the cluster. The shape of the cluster was optimized using the *Gaussian 16* program package (Frisch *et al.*, 2016 ▸) and the wB97XD functional (Chai & Head-Gordon, 2008 ▸). A polarized basis set with double-ζ quality (Def2-SVP) was employed for all atoms except Ir. For the latter, a relativistic effective core potential that includes 60 electrons in the core (ECP60MDF) was used, in combination with the ECP60MDF_VTZ valence basis set (Figgen *et al.*, 2009 ▸). An ultrafine integration grid was used throughout. The DFT-calculated lowest energy shape revealed average Ir—Ir bond lengths of 2.969 ± 0.142 Å, which are broadly consistent with those refined on the basis of X-ray data (2.944 ± 0.05 Å). DFT-calculated bond lengths within the penta­nuclear core are comparable to those within the crystal structure and differ by less than 6% (Fig. 5 ▸). The exception is the Ir1—Ir2 bond length, which is predicted by DFT to be longer [3.240 Å compared to 2.9682 (3) Å, reflecting a 9% difference)] The bond lengths for the DFT-calculated structure are given in Table 1 ▸ for comparison. Accordingly, the hydride ligands were initially placed on basis of the DFT-optimized structure, and were then included in the model. The hydride locations gave Ir—H bond lengths within 4% of the DFT predicted values. For final refinement, DFIX commands were used to restrain Ir—H bond lengths to be meaningful; the *U*_iso_ parameter of some of the hydride H atoms were refined freely and some were fixed at 0.04 or 0.05 Å^2^. We note that this placement is also consistent with hydride sites in analogous trimeric and tetra­meric Ir hydride clusters (Fig. 4 ▸) and generally gives an octa­hedral, or distorted octa­hedral, shape around each Ir atom. C-bound H atoms were refined with a riding model. Mesityl group atoms C30:C31, C29*A*:C29*B*, C28*A*:C28*B*, C32*A*:C32*B* are disordered over two sets of sites (refined ratio 0.57:0.43); the ADPs of these equivalent atoms were constrained to be equal.

## Supplementary Material

Crystal structure: contains datablock(s) I. DOI: 10.1107/S2056989025008023/wm5764sup1.cif

Structure factors: contains datablock(s) I. DOI: 10.1107/S2056989025008023/wm5764Isup3.hkl

CCDC reference: 2486492

Additional supporting information:  crystallographic information; 3D view; checkCIF report

Additional supporting information:  crystallographic information; 3D view; checkCIF report

## Figures and Tables

**Table 1:** Key bond lengths (Å) within the title compound

Bond type	Atom	Atom	Length determined by X-ray study	Length predicted by DFT study
Metal–Metal	Ir1	Ir3	3.0067 (3)	2.849
	Ir1	Ir4	2.9936 (3)	2.995
	Ir1	Ir5	2.9838 (3)	3.159
	Ir1	Ir2	2.9682 (3)	3.240
	Ir4	Ir5	2.9310 (3)	2.957
	Ir2	Ir4	2.9295 (3)	2.926
	Ir3	Ir5	2.9277 (3)	2.847
	Ir2	Ir3	2.9192 (3)	2.854
	Ir3	Ir4	2.8438 (3)	2.894
Metal–IMes	Ir1	C1	2.056 (5)	2.007
	Ir4	C44	2.007 (5)	2.003
	Ir5	C22	1.974 (5)	1.940
	Ir2	C65	1.950 (5)	1.944
Metal–CO	Ir3	C97	1.750 (6)	1.846
Metal–μ3 hydride	Ir3	H*A*	1.891	1.894
Metal–μ2 hydride	Ir5	H*K*	1.889	1.862
Metal–μ3 hydride	Ir3	H	1.889	1.895
Metal–μ3 hydride	Ir2	H*A*	1.888	1.901
Metal–μ3 hydride	Ir5	H	1.884	1.880
Metal–μ3 hydride	Ir1	H*A*	1.883	1.965
Metal–μ2 hydride	Ir2	H*A*	1.882	1.911
Metal–μ3 hydride	Ir1	H	1.879	1.930
Metal–μ2 hydride	Ir3	H*H*	1.754	1.761
Metal–μ2 hydride	Ir1	H*B*	1.748	1.739
Metal–μ2 hydride	Ir4	H*B*	1.742	1.778
Metal–μ2 hydride	Ir4	H*G*	1.706	1.707
Metal–μ2 hydride	Ir4	H*K*	1.702	1.712
Metal–μ2 hydride	Ir4	H*H*	1.697	1.698
Metal–μ1 hydride	Ir3	H*J*	1.661	1.894
Metal–μ1 hydride	Ir3	H*I*	1.660	1.586
Metal–μ1 hydride	Ir4	H*L*	1.596	1.597
Metal–μ1 hydride	Ir5	H*N*	1.590	1.592
Metal–μ1 hydride	Ir2	H*E*	1.582	1.578
Metal–μ1 hydride	Ir1	H*C*	1.564	1.576
Metal–μ1 hydride	Ir1	H*D*	1.562	1.559
Metal–μ1 hydride	Ir2	H*F*	1.535	1.535
Metal–μ1 hydride	Ir5	H*M*	1.527	1.534

**Table 2:** Experimental details

Crystal data	
Chemical formula	[Ir_5_H_15_(C_21_H_24_N_2_)_4_(CO)]
*M*_r_	2221.81
Crystal system, space group	Monoclinic, *P*2_1_/*c*
Temperature (K)	110
*a*, *b*, *c* (Å)	16.9589 (2), 15.7640 (2), 29.5834 (3)
β (°)	94.602 (1)
*V* (Å^3^)	7883.33 (16)
*Z*	4
Radiation type	Cu *K*α
μ (mm^−1^)	16.31
Crystal size (mm)	0.09 × 0.07 × 0.05

Data collection	
Diffractometer	Oxford Diffraction Supernova
Absorption correction	Gaussian (*CrysAlis PRO*; Rigaku OD, 2020 ▸)
*T*_min_, *T*_max_	0.918, 1.000
No. of measured, independent and observed [*I* > 2σ(*I*)] reflections	58807, 14393, 12837
*R*_int_	0.034
(sin θ/λ)_max_ (Å^−1^)	0.602

Refinement	
*R*[*F*^2^ > 2σ(*F*^2^)], *wR*(*F*^2^), *S*	0.028, 0.074, 1.04
No. of reflections	14393
No. of parameters	985
No. of restraints	36
H-atom treatment	H atoms treated by a mixture of independent and constrained refinement
Δρ_max_, Δρ_min_ (e Å^−3^)	3.41, −1.38

Computer programs: *CrysAlis PRO* (Rigaku OD, 2020 ▸), *SHELXL2018/3* (Sheldrick, 2015*a* ▸), *SHELXL2018/3* (Sheldrick, 2015*b* ▸), *OLEX2* (Dolomanov *et al.*, 2009 ▸) and *publCIF* (Westrip, 2010 ▸).

## supplementary crystallographic information

### Tetrakis[1,3-bis(2,4,6-trimethylphenyl)-1,3-dihydro-2H-imidazol-2-ylidene-κC2]carbonyldi-µ3-hydrido-tetra-µ2-hydrido-nonahydridopentairidium(III) Crystal data

**Table:**

[Ir_5_H_15_(C_21_H_24_N_2_)_4_(CO)]	*F*(000) = 4280
*M**_r_* = 2221.81	*D*_x_ = 1.872 Mg m^−^^3^
Monoclinic, *P*2_1_/*c*	Cu *K*α radiation, λ = 1.54184 Å
*a* = 16.9589 (2) Å	Cell parameters from 58809 reflections
*b* = 15.7640 (2) Å	θ = 5.2–68.3°
*c* = 29.5834 (3) Å	µ = 16.31 mm^−^^1^
β = 94.602 (1)°	*T* = 110 K
*V* = 7883.33 (16) Å^3^	Block, orange
*Z* = 4	0.09 × 0.07 × 0.05 mm

### Tetrakis[1,3-bis(2,4,6-trimethylphenyl)-1,3-dihydro-2H-imidazol-2-ylidene-κC2]carbonyldi-µ3-hydrido-tetra-µ2-hydrido-nonahydridopentairidium(III) Data collection

**Table:**

Oxford Diffraction Supernova diffractometer	12837 reflections with *I* > 2σ(*I*)
Radiation source: micro-focus sealed X-ray tube	*R*_int_ = 0.034
ω scans	θ_max_ = 68.3°, θ_min_ = 2.6°
Absorption correction: gaussian (CrysAlis PRO; Rigaku OD, 2020)	*h* = −20→20
*T*_min_ = 0.918, *T*_max_ = 1.000	*k* = −18→18
58807 measured reflections	*l* = −30→35
14393 independent reflections	

### Tetrakis[1,3-bis(2,4,6-trimethylphenyl)-1,3-dihydro-2H-imidazol-2-ylidene-κC2]carbonyldi-µ3-hydrido-tetra-µ2-hydrido-nonahydridopentairidium(III) Refinement

**Table:**

Refinement on *F*^2^	36 restraints
Least-squares matrix: full	Hydrogen site location: mixed
*R*[*F*^2^ > 2σ(*F*^2^)] = 0.028	H atoms treated by a mixture of independent and constrained refinement
*wR*(*F*^2^) = 0.074	*w* = 1/[σ^2^(*F*_o_^2^) + (0.0387*P*)^2^ + 24.9559*P*] where *P* = (*F*_o_^2^ + 2*F*_c_^2^)/3
*S* = 1.04	(Δ/σ)_max_ = 0.002
14393 reflections	Δρ_max_ = 3.41 e Å^−^^3^
985 parameters	Δρ_min_ = −1.37 e Å^−^^3^

### Tetrakis[1,3-bis(2,4,6-trimethylphenyl)-1,3-dihydro-2H-imidazol-2-ylidene-κC2]carbonyldi-µ3-hydrido-tetra-µ2-hydrido-nonahydridopentairidium(III) Special details

**Table:**

Geometry. All esds (except the esd in the dihedral angle between two l.s. planes) are estimated using the full covariance matrix. The cell esds are taken into account individually in the estimation of esds in distances, angles and torsion angles; correlations between esds in cell parameters are only used when they are defined by crystal symmetry. An approximate (isotropic) treatment of cell esds is used for estimating esds involving l.s. planes.
Refinement. Recorded dmax 3.4 and dmin -1.4. Split methyl group and part imes ligand atoms C30:C31, C29A:C29B, C28A:C28B, C32A:C32B modelled in the ratio of 0.57:0.43. ADP?s of these equivalence atoms are constrained to be equal.Then below is the list of DFIX and DANG for the bond length fixes and angle restraints for the hydrides and Iridiums as modelled from your DFT model. DFIX 1.59 0.01 O1 C86 FLAT 0.01 H HA Ir3 HI HJ DFIX 1.9 0.01 Ir3 HA Ir1 HA Ir2 HA DFIX 1.535 0.01 Ir5 HM Ir2 HF DANG 2.49 0.01 HM C22 HF C65 DANG 2.21 0.01 HN HM DFIX 1.71 0.01 Ir4 HG Ir4 HK DFIX 1.9 0.01 Ir5 H Ir3 H Ir1 H DFIX 1.89 0.01 Ir5 HK Ir2 HG DFIX 1.6 0.01 Ir3 HJ Ir3 HI DANG 3.33 0.02 Ir2 HJ HI Ir5 DANG 2.52 0.01 HN C22 HE C65 DANG 2.26 0.02 HJ HI DANG 2.21 0.01 HE HF DFIX 1.59 0.01 HE Ir2 HN Ir5 DFIX 1.76 0.01 Ir3 HH DFIX 1.7 0.01 Ir4 HH DFIX 1.6 0.01 Ir4 HL DFIX 1.75 0.01 Ir4 HB Ir1 HB DFIX 1.57 0.01 Ir1 HC Ir1 HD DANG 2.22 0.02 HC HD

### Tetrakis[1,3-bis(2,4,6-trimethylphenyl)-1,3-dihydro-2H-imidazol-2-ylidene-κC2]carbonyldi-µ3-hydrido-tetra-µ2-hydrido-nonahydridopentairidium(III) Fractional atomic coordinates and isotropic or equivalent isotropic displacement parameters (Å^2^)

**Table:**

	*x*	*y*	*z*	*U*_iso_*/*U*_eq_	Occ. (<1)
C1	0.0515 (3)	0.7049 (3)	0.38526 (17)	0.0250 (10)	
C2	−0.0775 (3)	0.7225 (4)	0.4013 (2)	0.0348 (12)	
H2	−0.122232	0.739184	0.416586	0.042*	
C3	−0.0782 (3)	0.6810 (4)	0.36205 (19)	0.0350 (12)	
H3	−0.123756	0.662574	0.343936	0.042*	
C4	0.0156 (3)	0.6212 (3)	0.31284 (16)	0.0258 (10)	
C5	0.0097 (3)	0.6613 (3)	0.27085 (18)	0.0295 (11)	
C6	−0.0076 (4)	0.7551 (4)	0.2670 (2)	0.0379 (13)	
H6A	−0.061374	0.766124	0.275422	0.057*	
H6B	−0.003002	0.773636	0.235706	0.057*	
H6C	0.030382	0.786443	0.287400	0.057*	
C7	0.0157 (3)	0.6115 (3)	0.23237 (18)	0.0313 (11)	
H7	0.013928	0.637895	0.203436	0.038*	
C8	0.0243 (3)	0.5240 (3)	0.23549 (17)	0.0302 (11)	
C9	0.0258 (4)	0.4708 (4)	0.19287 (17)	0.0353 (12)	
H9A	0.064850	0.425291	0.197943	0.053*	
H9B	0.040024	0.506652	0.167724	0.053*	
H9C	−0.026670	0.446024	0.185400	0.053*	
C10	0.0288 (3)	0.4867 (3)	0.27802 (17)	0.0310 (11)	
H10	0.034839	0.426951	0.280328	0.037*	
C11	0.0247 (3)	0.5339 (3)	0.31738 (16)	0.0278 (11)	
C12	0.0279 (4)	0.4917 (4)	0.36312 (18)	0.0357 (12)	
H12A	0.081911	0.494696	0.377499	0.054*	
H12B	0.012037	0.432148	0.359409	0.054*	
H12C	−0.008267	0.520710	0.382239	0.054*	
C13	0.0221 (3)	0.7694 (3)	0.46061 (17)	0.0276 (11)	
C14	0.0123 (3)	0.7142 (4)	0.49718 (19)	0.0335 (12)	
C15	−0.0110 (4)	0.6238 (4)	0.4892 (2)	0.0483 (16)	
H15A	0.028796	0.595361	0.472342	0.072*	
H15B	−0.062487	0.621429	0.471712	0.072*	
H15C	−0.014550	0.595309	0.518440	0.072*	
C16	0.0265 (3)	0.7466 (4)	0.54068 (19)	0.0362 (13)	
H16	0.018230	0.711015	0.565778	0.043*	
C17	0.0521 (3)	0.8279 (4)	0.54875 (18)	0.0357 (13)	
C18	0.0763 (4)	0.8581 (5)	0.59661 (19)	0.0468 (16)	
H18A	0.053993	0.819948	0.618450	0.070*	
H18B	0.056298	0.915692	0.600621	0.070*	
H18C	0.134085	0.858030	0.601638	0.070*	
C19	0.0580 (3)	0.8817 (4)	0.51209 (18)	0.0331 (12)	
H19	0.073764	0.938906	0.517495	0.040*	
C20	0.0414 (3)	0.8540 (4)	0.46712 (17)	0.0290 (11)	
C21	0.0438 (3)	0.9157 (3)	0.42840 (19)	0.0331 (12)	
H21A	0.097046	0.916438	0.417777	0.050*	
H21B	0.030328	0.972630	0.438716	0.050*	
H21C	0.005562	0.898346	0.403530	0.050*	
C22	0.2909 (3)	0.9881 (3)	0.35647 (17)	0.0265 (10)	
C23	0.3352 (3)	1.1233 (4)	0.3692 (2)	0.0385 (13)	
H23	0.347529	1.175784	0.383859	0.046*	
C24	0.3485 (4)	1.1022 (4)	0.3268 (2)	0.0441 (15)	
H24	0.372351	1.137427	0.305675	0.053*	
C25	0.3145 (3)	0.9831 (3)	0.27437 (18)	0.0337 (12)	
C26	0.2488 (4)	1.0052 (4)	0.2459 (2)	0.0447 (15)	
C27	0.1835 (4)	1.0615 (5)	0.2617 (2)	0.0515 (17)	
H27A	0.142261	1.068985	0.236859	0.077*	
H27B	0.205640	1.116927	0.270792	0.077*	
H27C	0.160703	1.034856	0.287552	0.077*	
C28A	0.2571 (13)	0.9858 (13)	0.2019 (8)	0.042 (3)	0.431 (9)
H28A	0.220089	1.008416	0.179283	0.050*	0.431 (9)
C29A	0.3200 (10)	0.9322 (12)	0.1880 (6)	0.041 (2)	0.431 (9)
C28B	0.2315 (10)	0.9601 (9)	0.2036 (6)	0.042 (3)	0.569 (9)
H28B	0.182446	0.968267	0.186242	0.050*	0.569 (9)
C29B	0.2860 (8)	0.9061 (9)	0.1890 (4)	0.041 (2)	0.569 (9)
C30	0.3220 (12)	0.9076 (14)	0.1382 (5)	0.064 (3)	0.431 (9)
H30A	0.275569	0.931189	0.120753	0.097*	0.431 (9)
H30B	0.370204	0.930151	0.126474	0.097*	0.431 (9)
H30C	0.321549	0.845644	0.135444	0.097*	0.431 (9)
C31	0.2715 (9)	0.8583 (10)	0.1456 (4)	0.064 (3)	0.569 (9)
H31A	0.317552	0.822725	0.140907	0.097*	0.569 (9)
H31B	0.224618	0.822432	0.146946	0.097*	0.569 (9)
H31C	0.262999	0.898488	0.120377	0.097*	0.569 (9)
C32A	0.3797 (16)	0.906 (2)	0.2183 (13)	0.039 (4)	0.431 (9)
H32A	0.422931	0.872828	0.209484	0.047*	0.431 (9)
C32B	0.3543 (10)	0.8900 (17)	0.2179 (9)	0.039 (4)	0.569 (9)
H32B	0.391070	0.850081	0.207912	0.047*	0.569 (9)
C33	0.3730 (4)	0.9289 (4)	0.26137 (18)	0.0355 (12)	
C34	0.4408 (3)	0.9035 (4)	0.2936 (2)	0.0412 (14)	
H34A	0.470117	0.857411	0.280361	0.062*	
H34B	0.420973	0.884173	0.322083	0.062*	
H34C	0.475964	0.952243	0.299646	0.062*	
C35	0.2694 (3)	1.0581 (3)	0.43181 (17)	0.0272 (11)	
C36	0.3177 (3)	1.0353 (3)	0.46995 (18)	0.0302 (11)	
C37	0.3978 (3)	0.9976 (4)	0.4655 (2)	0.0381 (13)	
H37A	0.392125	0.944018	0.448731	0.057*	
H37B	0.423842	0.986915	0.495746	0.057*	
H37C	0.429822	1.037107	0.449124	0.057*	
C38	0.2874 (3)	1.0443 (3)	0.51201 (18)	0.0323 (12)	
H38	0.319741	1.029094	0.538483	0.039*	
C39	0.2126 (3)	1.0743 (3)	0.51663 (18)	0.0302 (11)	
C40	0.1820 (4)	1.0829 (4)	0.56319 (19)	0.0400 (13)	
H40A	0.210017	1.129106	0.579774	0.060*	
H40B	0.190975	1.029682	0.579956	0.060*	
H40C	0.125211	1.095356	0.559916	0.060*	
C41	0.1650 (3)	1.0964 (3)	0.47757 (18)	0.0310 (11)	
H41	0.112839	1.116707	0.480266	0.037*	
C42	0.1932 (3)	1.0889 (3)	0.43464 (18)	0.0295 (11)	
C43	0.1414 (3)	1.1116 (4)	0.39243 (19)	0.0339 (12)	
H43A	0.123630	1.059556	0.376584	0.051*	
H43B	0.171543	1.146294	0.372424	0.051*	
H43C	0.095263	1.143608	0.400923	0.051*	
C44	0.4186 (3)	0.7403 (3)	0.43611 (16)	0.0249 (10)	
C45	0.4670 (3)	0.7640 (4)	0.50943 (18)	0.0323 (12)	
H45	0.469595	0.767666	0.541564	0.039*	
C46	0.5246 (3)	0.7837 (4)	0.48357 (17)	0.0307 (11)	
H46	0.575612	0.804302	0.493659	0.037*	
C47	0.5515 (3)	0.7692 (4)	0.40386 (17)	0.0284 (11)	
C48	0.5750 (3)	0.8464 (4)	0.38656 (17)	0.0302 (11)	
C49	0.5372 (3)	0.9280 (4)	0.3995 (2)	0.0353 (12)	
H49A	0.480655	0.926690	0.389652	0.053*	
H49B	0.562270	0.975604	0.384915	0.053*	
H49C	0.543949	0.935042	0.432544	0.053*	
C50	0.6348 (3)	0.8460 (4)	0.35646 (18)	0.0335 (12)	
H50	0.651323	0.898097	0.344179	0.040*	
C51	0.6706 (3)	0.7703 (4)	0.34411 (18)	0.0352 (13)	
C52	0.7367 (4)	0.7711 (5)	0.3126 (2)	0.0481 (16)	
H52A	0.782948	0.800109	0.327434	0.072*	
H52B	0.719050	0.801057	0.284583	0.072*	
H52C	0.750994	0.712673	0.305418	0.072*	
C53	0.6447 (3)	0.6959 (4)	0.36180 (19)	0.0367 (13)	
H53	0.667933	0.644243	0.353019	0.044*	
C54	0.5859 (3)	0.6923 (4)	0.39194 (18)	0.0314 (11)	
C55	0.5604 (3)	0.6094 (4)	0.4107 (2)	0.0382 (13)	
H55A	0.591511	0.563387	0.398661	0.057*	
H55B	0.504186	0.600264	0.401743	0.057*	
H55C	0.568951	0.610388	0.443807	0.057*	
C56	0.3366 (3)	0.6926 (3)	0.49852 (15)	0.0280 (11)	
C57	0.3376 (3)	0.6041 (4)	0.49795 (17)	0.0332 (12)	
C58	0.4030 (4)	0.5570 (4)	0.47764 (19)	0.0410 (14)	
H58A	0.393846	0.557547	0.444506	0.062*	
H58B	0.404308	0.498275	0.488496	0.062*	
H58C	0.453713	0.584523	0.486623	0.062*	
C59	0.2772 (4)	0.5613 (4)	0.51666 (19)	0.0387 (13)	
H59	0.276242	0.501061	0.515460	0.046*	
C60	0.2179 (4)	0.6035 (4)	0.5372 (2)	0.0416 (14)	
C61	0.1551 (4)	0.5535 (5)	0.5590 (3)	0.061 (2)	
H61A	0.121591	0.523931	0.535461	0.091*	
H61B	0.122482	0.592252	0.575511	0.091*	
H61C	0.180338	0.511979	0.580103	0.091*	
C62	0.2194 (3)	0.6918 (4)	0.53773 (19)	0.0366 (13)	
H62	0.179428	0.721461	0.551998	0.044*	
C63	0.2781 (3)	0.7383 (4)	0.51787 (16)	0.0304 (11)	
C64	0.2771 (3)	0.8334 (4)	0.51712 (18)	0.0333 (12)	
H64A	0.328686	0.855059	0.529260	0.050*	
H64B	0.235911	0.854164	0.535751	0.050*	
H64C	0.266006	0.853203	0.485849	0.050*	
C65	0.3197 (3)	0.4845 (3)	0.31759 (16)	0.0263 (10)	
C66	0.3656 (3)	0.3510 (4)	0.30284 (18)	0.0353 (12)	
H66	0.377530	0.292379	0.306171	0.042*	
C67	0.3817 (3)	0.4005 (3)	0.26822 (18)	0.0333 (12)	
H67	0.407602	0.383677	0.242364	0.040*	
C68	0.3641 (3)	0.5491 (3)	0.24567 (16)	0.0265 (10)	
C69	0.4345 (3)	0.5955 (4)	0.25012 (18)	0.0313 (11)	
C70	0.4907 (3)	0.5877 (4)	0.29208 (19)	0.0397 (13)	
H70A	0.538194	0.621498	0.288313	0.060*	
H70B	0.505539	0.528068	0.296790	0.060*	
H70C	0.464809	0.608424	0.318395	0.060*	
C71	0.3096 (3)	0.5586 (4)	0.20829 (17)	0.0310 (11)	
C72	0.2306 (4)	0.5147 (4)	0.2060 (2)	0.0416 (14)	
H72A	0.194082	0.547189	0.223320	0.062*	
H72B	0.237095	0.457567	0.218853	0.062*	
H72C	0.209197	0.510629	0.174294	0.062*	
C73	0.3298 (4)	0.6119 (4)	0.17343 (18)	0.0386 (13)	
H73	0.293757	0.618552	0.147379	0.046*	
C74	0.4005 (4)	0.6550 (4)	0.17574 (19)	0.0425 (14)	
C75	0.4258 (5)	0.7059 (5)	0.1357 (2)	0.060 (2)	
H75A	0.451698	0.758369	0.146768	0.091*	
H75B	0.379155	0.719867	0.115375	0.091*	
H75C	0.462796	0.672311	0.119293	0.091*	
C76	0.4509 (3)	0.6491 (4)	0.21507 (19)	0.0387 (13)	
H76	0.497646	0.682525	0.217933	0.046*	
C77	0.3062 (3)	0.3692 (3)	0.37590 (17)	0.0266 (10)	
C78	0.2307 (3)	0.3381 (3)	0.37919 (17)	0.0308 (11)	
C79	0.1727 (4)	0.3311 (4)	0.3384 (2)	0.0383 (13)	
H79A	0.160351	0.387840	0.326296	0.057*	
H79B	0.124150	0.303973	0.347019	0.057*	
H79C	0.195760	0.296800	0.315136	0.057*	
C80	0.2102 (4)	0.3136 (3)	0.4223 (2)	0.0354 (12)	
H80	0.158261	0.293268	0.425798	0.043*	
C81	0.2647 (4)	0.3185 (3)	0.46012 (18)	0.0345 (12)	
C82	0.2405 (4)	0.2975 (4)	0.5071 (2)	0.0465 (15)	
H82A	0.275013	0.252858	0.520645	0.070*	
H82B	0.185502	0.277726	0.504922	0.070*	
H82C	0.245260	0.348347	0.526224	0.070*	
C83	0.3412 (3)	0.3446 (4)	0.45401 (18)	0.0344 (12)	
H83	0.379424	0.344475	0.479306	0.041*	
C84	0.3638 (3)	0.3708 (3)	0.41210 (18)	0.0297 (11)	
C85	0.4464 (3)	0.4015 (4)	0.4058 (2)	0.0374 (13)	
H85A	0.477344	0.402619	0.435212	0.056*	
H85B	0.443924	0.458680	0.392818	0.056*	
H85C	0.471522	0.362998	0.385249	0.056*	
C86	0.1777 (4)	0.7470 (4)	0.2500 (2)	0.0428 (14)	
Ir1	0.17303 (2)	0.70969 (2)	0.38949 (2)	0.02179 (6)	
H	0.1762 (8)	0.7883 (6)	0.3419 (3)	0.37 (14)*	
HA	0.1851 (7)	0.6586 (6)	0.3329 (3)	0.040*	
HB	0.2686 (13)	0.727 (10)	0.4144 (8)	0.22 (7)*	
HC	0.152 (4)	0.6189 (14)	0.4061 (16)	0.053 (19)*	
HD	0.156 (3)	0.749 (3)	0.4360 (9)	0.033 (15)*	
Ir2	0.26399 (2)	0.57580 (2)	0.34597 (2)	0.02314 (6)	
HE	0.1818 (5)	0.5387 (19)	0.3260 (13)	0.044 (17)*	
HF	0.260 (2)	0.5220 (15)	0.3890 (6)	0.049 (18)*	
HG	0.3573 (14)	0.6226 (8)	0.3745 (18)	0.040 (17)*	
Ir3	0.24045 (2)	0.73859 (2)	0.29981 (2)	0.02245 (6)	
HH	0.3394 (7)	0.741 (4)	0.3221 (4)	0.035 (16)*	
HI	0.2862 (9)	0.8138 (11)	0.2726 (4)	0.36 (13)*	
HJ	0.2947 (8)	0.6884 (12)	0.2639 (4)	0.29 (10)*	
Ir4	0.34624 (2)	0.72971 (2)	0.37927 (2)	0.02304 (6)	
HK	0.3429 (13)	0.8368 (8)	0.386 (2)	0.050*	
HL	0.431 (2)	0.731 (5)	0.360 (3)	0.08 (3)*	
Ir5	0.24133 (2)	0.87687 (2)	0.36592 (2)	0.02145 (6)	
HM	0.223 (2)	0.9049 (14)	0.4134 (5)	0.050*	
HN	0.1591 (7)	0.9145 (13)	0.3454 (12)	0.017 (12)*	
N1	−0.0004 (2)	0.6698 (3)	0.35249 (14)	0.0281 (9)	
N2	0.0022 (2)	0.7366 (3)	0.41563 (14)	0.0271 (9)	
N3	0.3284 (3)	0.4007 (3)	0.33273 (14)	0.0272 (9)	
N4	0.3536 (3)	0.4812 (3)	0.27694 (14)	0.0276 (9)	
N5	0.3214 (2)	1.0203 (3)	0.31892 (14)	0.0274 (9)	
N6	0.3001 (2)	1.0537 (3)	0.38769 (14)	0.0272 (9)	
N7	0.4961 (2)	0.7684 (3)	0.43874 (14)	0.0262 (9)	
N8	0.4017 (2)	0.7368 (3)	0.48128 (13)	0.0258 (9)	
O1	0.1418 (3)	0.7546 (3)	0.20983 (13)	0.0525 (12)	

### Tetrakis[1,3-bis(2,4,6-trimethylphenyl)-1,3-dihydro-2H-imidazol-2-ylidene-κC2]carbonyldi-µ3-hydrido-tetra-µ2-hydrido-nonahydridopentairidium(III) Atomic displacement parameters (Å^2^)

**Table:**

	*U*^11^	*U*^22^	*U*^33^	*U*^12^	*U*^13^	*U*^23^
C1	0.026 (2)	0.020 (2)	0.029 (2)	0.001 (2)	−0.0019 (19)	−0.003 (2)
C2	0.023 (3)	0.039 (3)	0.042 (3)	0.003 (2)	0.003 (2)	−0.008 (3)
C3	0.026 (3)	0.037 (3)	0.041 (3)	0.000 (2)	−0.004 (2)	−0.011 (3)
C4	0.023 (2)	0.028 (3)	0.026 (2)	−0.001 (2)	−0.0011 (19)	−0.007 (2)
C5	0.028 (3)	0.023 (3)	0.035 (3)	−0.002 (2)	−0.008 (2)	−0.003 (2)
C6	0.047 (3)	0.026 (3)	0.039 (3)	0.008 (3)	−0.009 (3)	−0.001 (2)
C7	0.035 (3)	0.029 (3)	0.028 (3)	−0.006 (2)	−0.004 (2)	0.001 (2)
C8	0.033 (3)	0.029 (3)	0.028 (3)	−0.007 (2)	−0.002 (2)	−0.006 (2)
C9	0.049 (3)	0.030 (3)	0.026 (3)	−0.008 (3)	0.002 (2)	−0.002 (2)
C10	0.037 (3)	0.023 (3)	0.032 (3)	−0.004 (2)	0.000 (2)	−0.001 (2)
C11	0.027 (2)	0.031 (3)	0.026 (2)	−0.002 (2)	0.0003 (19)	−0.002 (2)
C12	0.044 (3)	0.033 (3)	0.031 (3)	−0.003 (3)	0.005 (2)	0.000 (2)
C13	0.027 (2)	0.031 (3)	0.025 (2)	0.001 (2)	0.002 (2)	−0.007 (2)
C14	0.028 (3)	0.037 (3)	0.036 (3)	−0.004 (2)	0.006 (2)	−0.003 (2)
C15	0.053 (4)	0.041 (4)	0.051 (4)	−0.008 (3)	0.000 (3)	0.003 (3)
C16	0.028 (3)	0.048 (3)	0.034 (3)	0.005 (3)	0.007 (2)	0.001 (3)
C17	0.031 (3)	0.047 (3)	0.030 (3)	0.006 (3)	0.008 (2)	−0.007 (3)
C18	0.043 (3)	0.065 (4)	0.033 (3)	0.009 (3)	0.005 (2)	−0.012 (3)
C19	0.029 (3)	0.035 (3)	0.036 (3)	0.000 (2)	0.009 (2)	−0.010 (2)
C20	0.022 (2)	0.034 (3)	0.032 (3)	0.004 (2)	0.008 (2)	−0.006 (2)
C21	0.033 (3)	0.028 (3)	0.041 (3)	−0.003 (2)	0.013 (2)	−0.004 (2)
C22	0.020 (2)	0.028 (3)	0.031 (3)	−0.001 (2)	0.0039 (19)	−0.002 (2)
C23	0.042 (3)	0.027 (3)	0.048 (3)	−0.014 (2)	0.016 (3)	−0.007 (3)
C24	0.044 (3)	0.032 (3)	0.059 (4)	−0.017 (3)	0.018 (3)	0.000 (3)
C25	0.042 (3)	0.025 (3)	0.035 (3)	−0.005 (2)	0.011 (2)	0.005 (2)
C26	0.056 (4)	0.043 (4)	0.036 (3)	0.009 (3)	0.007 (3)	0.006 (3)
C27	0.054 (4)	0.052 (4)	0.049 (4)	0.013 (3)	0.004 (3)	0.010 (3)
C28A	0.058 (10)	0.028 (9)	0.037 (4)	−0.009 (5)	−0.006 (6)	0.009 (6)
C29A	0.041 (8)	0.053 (8)	0.032 (4)	−0.004 (5)	0.004 (5)	−0.006 (5)
C28B	0.058 (10)	0.028 (9)	0.037 (4)	−0.009 (5)	−0.006 (6)	0.009 (6)
C29B	0.041 (8)	0.053 (8)	0.032 (4)	−0.004 (5)	0.004 (5)	−0.006 (5)
C30	0.074 (8)	0.084 (9)	0.035 (5)	0.006 (6)	0.001 (5)	−0.005 (5)
C31	0.074 (8)	0.084 (9)	0.035 (5)	0.006 (6)	0.001 (5)	−0.005 (5)
C32A	0.039 (12)	0.045 (10)	0.036 (4)	−0.002 (8)	0.018 (9)	0.003 (6)
C32B	0.039 (12)	0.045 (10)	0.036 (4)	−0.002 (8)	0.018 (9)	0.003 (6)
C33	0.047 (3)	0.029 (3)	0.032 (3)	−0.002 (2)	0.015 (2)	0.002 (2)
C34	0.036 (3)	0.041 (3)	0.049 (3)	−0.002 (3)	0.013 (3)	−0.004 (3)
C35	0.026 (2)	0.023 (3)	0.033 (3)	−0.003 (2)	0.004 (2)	−0.010 (2)
C36	0.026 (2)	0.027 (3)	0.038 (3)	−0.002 (2)	0.004 (2)	−0.014 (2)
C37	0.030 (3)	0.045 (3)	0.039 (3)	0.001 (3)	0.002 (2)	−0.012 (3)
C38	0.030 (3)	0.031 (3)	0.035 (3)	−0.001 (2)	−0.003 (2)	−0.011 (2)
C39	0.032 (3)	0.028 (3)	0.031 (3)	−0.003 (2)	0.004 (2)	−0.010 (2)
C40	0.038 (3)	0.046 (4)	0.037 (3)	−0.001 (3)	0.005 (2)	−0.010 (3)
C41	0.026 (3)	0.026 (3)	0.042 (3)	0.000 (2)	0.007 (2)	−0.010 (2)
C42	0.026 (2)	0.025 (3)	0.037 (3)	−0.003 (2)	0.002 (2)	−0.006 (2)
C43	0.034 (3)	0.028 (3)	0.039 (3)	0.002 (2)	0.000 (2)	−0.003 (2)
C44	0.030 (3)	0.022 (2)	0.023 (2)	0.006 (2)	0.003 (2)	−0.0015 (19)
C45	0.032 (3)	0.036 (3)	0.027 (3)	0.002 (2)	−0.008 (2)	−0.005 (2)
C46	0.029 (3)	0.036 (3)	0.026 (3)	0.003 (2)	−0.005 (2)	0.000 (2)
C47	0.020 (2)	0.038 (3)	0.026 (2)	0.005 (2)	−0.0031 (19)	−0.002 (2)
C48	0.023 (2)	0.035 (3)	0.032 (3)	0.001 (2)	−0.004 (2)	−0.002 (2)
C49	0.030 (3)	0.034 (3)	0.042 (3)	−0.004 (2)	0.004 (2)	−0.001 (2)
C50	0.023 (2)	0.042 (3)	0.035 (3)	0.000 (2)	−0.001 (2)	0.003 (2)
C51	0.030 (3)	0.047 (4)	0.028 (3)	0.007 (2)	0.002 (2)	0.000 (2)
C52	0.042 (3)	0.062 (4)	0.041 (3)	0.017 (3)	0.013 (3)	0.007 (3)
C53	0.032 (3)	0.042 (3)	0.035 (3)	0.013 (3)	−0.002 (2)	−0.008 (3)
C54	0.026 (3)	0.037 (3)	0.030 (3)	0.007 (2)	−0.003 (2)	−0.001 (2)
C55	0.037 (3)	0.034 (3)	0.044 (3)	0.005 (2)	0.005 (2)	0.001 (3)
C56	0.033 (3)	0.034 (3)	0.015 (2)	0.001 (2)	−0.0055 (19)	0.001 (2)
C57	0.041 (3)	0.035 (3)	0.022 (2)	0.002 (2)	−0.007 (2)	0.004 (2)
C58	0.056 (4)	0.032 (3)	0.033 (3)	0.008 (3)	−0.003 (3)	0.002 (2)
C59	0.049 (3)	0.033 (3)	0.033 (3)	−0.001 (3)	−0.005 (2)	0.006 (2)
C60	0.039 (3)	0.043 (3)	0.041 (3)	−0.007 (3)	−0.007 (3)	0.012 (3)
C61	0.048 (4)	0.056 (5)	0.077 (5)	−0.014 (3)	0.003 (3)	0.025 (4)
C62	0.034 (3)	0.042 (3)	0.034 (3)	−0.003 (3)	0.000 (2)	0.004 (3)
C63	0.032 (3)	0.038 (3)	0.020 (2)	−0.001 (2)	−0.005 (2)	0.002 (2)
C64	0.030 (3)	0.037 (3)	0.033 (3)	0.002 (2)	0.004 (2)	0.000 (2)
C65	0.029 (2)	0.027 (3)	0.023 (2)	0.004 (2)	0.0018 (19)	0.001 (2)
C66	0.046 (3)	0.024 (3)	0.037 (3)	0.010 (2)	0.008 (2)	−0.003 (2)
C67	0.042 (3)	0.025 (3)	0.034 (3)	0.010 (2)	0.010 (2)	−0.006 (2)
C68	0.033 (3)	0.028 (3)	0.019 (2)	0.002 (2)	0.0069 (19)	0.001 (2)
C69	0.034 (3)	0.031 (3)	0.031 (3)	0.003 (2)	0.010 (2)	−0.005 (2)
C70	0.032 (3)	0.048 (4)	0.039 (3)	0.001 (3)	0.001 (2)	−0.004 (3)
C71	0.035 (3)	0.036 (3)	0.022 (2)	0.003 (2)	0.005 (2)	−0.005 (2)
C72	0.039 (3)	0.048 (4)	0.037 (3)	−0.001 (3)	0.000 (2)	−0.002 (3)
C73	0.047 (3)	0.044 (3)	0.025 (3)	0.009 (3)	0.006 (2)	0.003 (2)
C74	0.055 (4)	0.043 (4)	0.031 (3)	0.009 (3)	0.019 (3)	0.007 (3)
C75	0.073 (5)	0.065 (5)	0.046 (4)	0.000 (4)	0.023 (3)	0.021 (4)
C76	0.038 (3)	0.041 (3)	0.041 (3)	−0.003 (3)	0.021 (2)	−0.004 (3)
C77	0.032 (3)	0.019 (2)	0.028 (3)	0.007 (2)	0.002 (2)	0.000 (2)
C78	0.040 (3)	0.021 (3)	0.030 (3)	0.003 (2)	0.000 (2)	0.003 (2)
C79	0.043 (3)	0.025 (3)	0.045 (3)	−0.005 (2)	−0.005 (3)	0.004 (2)
C80	0.041 (3)	0.023 (3)	0.043 (3)	−0.003 (2)	0.006 (2)	0.003 (2)
C81	0.049 (3)	0.025 (3)	0.030 (3)	0.001 (2)	0.006 (2)	0.003 (2)
C82	0.066 (4)	0.039 (3)	0.036 (3)	−0.008 (3)	0.010 (3)	0.002 (3)
C83	0.043 (3)	0.030 (3)	0.029 (3)	0.007 (2)	−0.003 (2)	0.001 (2)
C84	0.037 (3)	0.017 (2)	0.035 (3)	0.003 (2)	0.002 (2)	0.002 (2)
C85	0.038 (3)	0.037 (3)	0.038 (3)	0.006 (3)	0.002 (2)	−0.006 (3)
C86	0.049 (4)	0.031 (3)	0.048 (4)	−0.001 (3)	0.002 (3)	0.001 (3)
Ir1	0.02286 (10)	0.02109 (11)	0.02144 (10)	−0.00026 (8)	0.00191 (8)	−0.00110 (8)
Ir2	0.02937 (11)	0.01928 (11)	0.02094 (10)	0.00278 (8)	0.00316 (8)	−0.00035 (8)
Ir3	0.02822 (11)	0.02114 (11)	0.01782 (10)	0.00049 (8)	0.00091 (8)	0.00011 (8)
Ir4	0.02503 (11)	0.02249 (11)	0.02087 (10)	0.00218 (8)	−0.00267 (8)	−0.00086 (8)
Ir5	0.02289 (10)	0.01867 (10)	0.02272 (10)	−0.00042 (8)	0.00136 (8)	−0.00023 (8)
N1	0.025 (2)	0.027 (2)	0.032 (2)	−0.0017 (18)	−0.0015 (17)	−0.0070 (18)
N2	0.023 (2)	0.029 (2)	0.029 (2)	−0.0012 (18)	0.0027 (17)	−0.0064 (18)
N3	0.035 (2)	0.019 (2)	0.028 (2)	0.0023 (18)	0.0052 (18)	−0.0013 (17)
N4	0.034 (2)	0.024 (2)	0.025 (2)	0.0048 (18)	0.0077 (17)	0.0004 (17)
N5	0.032 (2)	0.022 (2)	0.030 (2)	−0.0055 (18)	0.0122 (17)	−0.0014 (17)
N6	0.025 (2)	0.025 (2)	0.032 (2)	−0.0049 (17)	0.0069 (17)	−0.0060 (18)
N7	0.027 (2)	0.029 (2)	0.022 (2)	0.0005 (18)	−0.0013 (16)	0.0001 (17)
N8	0.029 (2)	0.028 (2)	0.020 (2)	0.0017 (18)	−0.0003 (16)	−0.0003 (17)
O1	0.063 (3)	0.062 (3)	0.029 (2)	−0.009 (2)	−0.014 (2)	0.007 (2)

### Tetrakis[1,3-bis(2,4,6-trimethylphenyl)-1,3-dihydro-2H-imidazol-2-ylidene-κC2]carbonyldi-µ3-hydrido-tetra-µ2-hydrido-nonahydridopentairidium(III) Geometric parameters (Å, º)

**Table:**

C1—Ir1	2.056 (5)	C48—C50	1.402 (8)
C1—N1	1.372 (6)	C49—H49A	0.9800
C1—N2	1.371 (6)	C49—H49B	0.9800
C2—H2	0.9500	C49—H49C	0.9800
C2—C3	1.332 (8)	C50—H50	0.9500
C2—N2	1.401 (7)	C50—C51	1.401 (8)
C3—H3	0.9500	C51—C52	1.514 (8)
C3—N1	1.383 (7)	C51—C53	1.371 (9)
C4—C5	1.390 (7)	C52—H52A	0.9800
C4—C11	1.389 (7)	C52—H52B	0.9800
C4—N1	1.445 (6)	C52—H52C	0.9800
C5—C6	1.510 (7)	C53—H53	0.9500
C5—C7	1.393 (8)	C53—C54	1.390 (8)
C6—H6A	0.9800	C54—C55	1.496 (8)
C6—H6B	0.9800	C55—H55A	0.9800
C6—H6C	0.9800	C55—H55B	0.9800
C7—H7	0.9500	C55—H55C	0.9800
C7—C8	1.389 (8)	C56—C57	1.397 (8)
C8—C9	1.516 (7)	C56—C63	1.385 (8)
C8—C10	1.385 (7)	C56—N8	1.434 (7)
C9—H9A	0.9800	C57—C58	1.499 (8)
C9—H9B	0.9800	C57—C59	1.379 (8)
C9—H9C	0.9800	C58—H58A	0.9800
C10—H10	0.9500	C58—H58B	0.9800
C10—C11	1.388 (7)	C58—H58C	0.9800
C11—C12	1.505 (7)	C59—H59	0.9500
C12—H12A	0.9800	C59—C60	1.386 (9)
C12—H12B	0.9800	C60—C61	1.511 (9)
C12—H12C	0.9800	C60—C62	1.392 (9)
C13—C14	1.409 (8)	C61—H61A	0.9800
C13—C20	1.383 (8)	C61—H61B	0.9800
C13—N2	1.442 (6)	C61—H61C	0.9800
C14—C15	1.492 (8)	C62—H62	0.9500
C14—C16	1.388 (8)	C62—C63	1.403 (8)
C15—H15A	0.9800	C63—C64	1.500 (8)
C15—H15B	0.9800	C64—H64A	0.9800
C15—H15C	0.9800	C64—H64B	0.9800
C16—H16	0.9500	C64—H64C	0.9800
C16—C17	1.368 (9)	C65—Ir2	1.949 (5)
C17—C18	1.518 (8)	C65—N3	1.399 (6)
C17—C19	1.387 (8)	C65—N4	1.375 (6)
C18—H18A	0.9800	C66—H66	0.9500
C18—H18B	0.9800	C66—C67	1.333 (8)
C18—H18C	0.9800	C66—N3	1.371 (7)
C19—H19	0.9500	C67—H67	0.9500
C19—C20	1.407 (7)	C67—N4	1.390 (7)
C20—C21	1.506 (8)	C68—C69	1.396 (8)
C21—H21A	0.9800	C68—C71	1.392 (7)
C21—H21B	0.9800	C68—N4	1.436 (6)
C21—H21C	0.9800	C69—C70	1.508 (8)
C22—Ir5	1.974 (5)	C69—C76	1.383 (8)
C22—N5	1.361 (6)	C70—H70A	0.9800
C22—N6	1.387 (6)	C70—H70B	0.9800
C23—H23	0.9500	C70—H70C	0.9800
C23—C24	1.334 (9)	C71—C72	1.504 (8)
C23—N6	1.382 (7)	C71—C73	1.394 (8)
C24—H24	0.9500	C72—H72A	0.9800
C24—N5	1.384 (7)	C72—H72B	0.9800
C25—C26	1.387 (9)	C72—H72C	0.9800
C25—C33	1.387 (8)	C73—H73	0.9500
C25—N5	1.439 (7)	C73—C74	1.376 (9)
C26—C27	1.520 (9)	C74—C75	1.519 (8)
C26—C28A	1.35 (3)	C74—C76	1.391 (9)
C26—C28B	1.449 (17)	C75—H75A	0.9800
C27—H27A	0.9800	C75—H75B	0.9800
C27—H27B	0.9800	C75—H75C	0.9800
C27—H27C	0.9800	C76—H76	0.9500
C28A—H28A	0.9500	C77—C78	1.381 (8)
C28A—C29A	1.45 (3)	C77—C84	1.390 (7)
C29A—C30	1.53 (2)	C77—N3	1.448 (7)
C29A—C32A	1.36 (4)	C78—C79	1.499 (7)
C28B—H28B	0.9500	C78—C80	1.404 (8)
C28B—C29B	1.35 (2)	C79—H79A	0.9800
C29B—C31	1.493 (17)	C79—H79B	0.9800
C29B—C32B	1.41 (3)	C79—H79C	0.9800
C30—H30A	0.9800	C80—H80	0.9500
C30—H30B	0.9800	C80—C81	1.395 (8)
C30—H30C	0.9800	C81—C82	1.518 (8)
C31—H31A	0.9800	C81—C83	1.387 (8)
C31—H31B	0.9800	C82—H82A	0.9800
C31—H31C	0.9800	C82—H82B	0.9800
C32A—H32A	0.9500	C82—H82C	0.9800
C32A—C33	1.34 (4)	C83—H83	0.9500
C32B—H32B	0.9500	C83—C84	1.389 (8)
C32B—C33	1.44 (3)	C84—C85	1.508 (8)
C33—C34	1.489 (8)	C85—H85A	0.9800
C34—H34A	0.9800	C85—H85B	0.9800
C34—H34B	0.9800	C85—H85C	0.9800
C34—H34C	0.9800	C86—Ir3	1.752 (6)
C35—C36	1.387 (7)	C86—O1	1.297 (6)
C35—C42	1.389 (7)	Ir1—H	1.879 (10)
C35—N6	1.445 (6)	Ir1—HA	1.884 (10)
C36—C37	1.499 (7)	Ir1—HB	1.748 (10)
C36—C38	1.391 (8)	Ir1—HC	1.564 (10)
C37—H37A	0.9800	Ir1—HD	1.561 (10)
C37—H37B	0.9800	Ir1—Ir2	2.9683 (3)
C37—H37C	0.9800	Ir1—Ir3	3.0067 (3)
C38—H38	0.9500	Ir1—Ir4	2.9937 (3)
C38—C39	1.372 (8)	Ir1—Ir5	2.9838 (3)
C39—C40	1.516 (8)	Ir2—HA	1.888 (10)
C39—C41	1.399 (8)	Ir2—HE	1.582 (10)
C40—H40A	0.9800	Ir2—HF	1.535 (10)
C40—H40B	0.9800	Ir2—HG	1.882 (10)
C40—H40C	0.9800	Ir2—Ir3	2.9193 (3)
C41—H41	0.9500	Ir2—Ir4	2.9295 (3)
C41—C42	1.398 (8)	Ir3—H	1.888 (10)
C42—C43	1.511 (7)	Ir3—HA	1.891 (10)
C43—H43A	0.9800	Ir3—HH	1.753 (10)
C43—H43B	0.9800	Ir3—HI	1.660 (10)
C43—H43C	0.9800	Ir3—HJ	1.660 (10)
C44—Ir4	2.007 (5)	Ir3—Ir4	2.8438 (3)
C44—N7	1.384 (7)	Ir3—Ir5	2.9278 (3)
C44—N8	1.390 (6)	Ir4—HB	1.742 (10)
C45—H45	0.9500	Ir4—HG	1.705 (10)
C45—C46	1.325 (8)	Ir4—HH	1.697 (10)
C45—N8	1.398 (7)	Ir4—HK	1.702 (10)
C46—H46	0.9500	Ir4—HL	1.596 (10)
C46—N7	1.395 (6)	Ir4—Ir5	2.9310 (3)
C47—C48	1.391 (8)	Ir5—H	1.884 (10)
C47—C54	1.402 (8)	Ir5—HK	1.889 (10)
C47—N7	1.450 (7)	Ir5—HM	1.527 (10)
C48—C49	1.502 (8)	Ir5—HN	1.590 (10)
			
N1—C1—Ir1	129.8 (4)	H70A—C70—H70B	109.5
N2—C1—Ir1	127.5 (3)	H70A—C70—H70C	109.5
N2—C1—N1	102.7 (4)	H70B—C70—H70C	109.5
C3—C2—H2	126.8	C68—C71—C72	121.3 (5)
C3—C2—N2	106.4 (5)	C68—C71—C73	117.7 (5)
N2—C2—H2	126.8	C73—C71—C72	121.0 (5)
C2—C3—H3	126.3	C71—C72—H72A	109.5
C2—C3—N1	107.5 (5)	C71—C72—H72B	109.5
N1—C3—H3	126.3	C71—C72—H72C	109.5
C5—C4—N1	118.6 (5)	H72A—C72—H72B	109.5
C11—C4—C5	122.4 (5)	H72A—C72—H72C	109.5
C11—C4—N1	118.2 (4)	H72B—C72—H72C	109.5
C4—C5—C6	120.8 (5)	C71—C73—H73	119.1
C4—C5—C7	118.0 (5)	C74—C73—C71	121.8 (5)
C7—C5—C6	121.1 (5)	C74—C73—H73	119.1
C5—C6—H6A	109.5	C73—C74—C75	121.4 (6)
C5—C6—H6B	109.5	C73—C74—C76	118.9 (5)
C5—C6—H6C	109.5	C76—C74—C75	119.6 (6)
H6A—C6—H6B	109.5	C74—C75—H75A	109.5
H6A—C6—H6C	109.5	C74—C75—H75B	109.5
H6B—C6—H6C	109.5	C74—C75—H75C	109.5
C5—C7—H7	119.4	H75A—C75—H75B	109.5
C8—C7—C5	121.3 (5)	H75A—C75—H75C	109.5
C8—C7—H7	119.4	H75B—C75—H75C	109.5
C7—C8—C9	120.2 (5)	C69—C76—C74	121.4 (6)
C10—C8—C7	118.6 (5)	C69—C76—H76	119.3
C10—C8—C9	121.1 (5)	C74—C76—H76	119.3
C8—C9—H9A	109.5	C78—C77—C84	123.3 (5)
C8—C9—H9B	109.5	C78—C77—N3	119.5 (4)
C8—C9—H9C	109.5	C84—C77—N3	117.2 (5)
H9A—C9—H9B	109.5	C77—C78—C79	121.4 (5)
H9A—C9—H9C	109.5	C77—C78—C80	117.5 (5)
H9B—C9—H9C	109.5	C80—C78—C79	121.1 (5)
C8—C10—H10	118.9	C78—C79—H79A	109.5
C8—C10—C11	122.1 (5)	C78—C79—H79B	109.5
C11—C10—H10	118.9	C78—C79—H79C	109.5
C4—C11—C12	121.4 (5)	H79A—C79—H79B	109.5
C10—C11—C4	117.5 (5)	H79A—C79—H79C	109.5
C10—C11—C12	121.1 (5)	H79B—C79—H79C	109.5
C11—C12—H12A	109.5	C78—C80—H80	119.4
C11—C12—H12B	109.5	C81—C80—C78	121.1 (5)
C11—C12—H12C	109.5	C81—C80—H80	119.4
H12A—C12—H12B	109.5	C80—C81—C82	120.8 (5)
H12A—C12—H12C	109.5	C83—C81—C80	118.6 (5)
H12B—C12—H12C	109.5	C83—C81—C82	120.6 (5)
C14—C13—N2	117.0 (5)	C81—C82—H82A	109.5
C20—C13—C14	122.0 (5)	C81—C82—H82B	109.5
C20—C13—N2	120.6 (5)	C81—C82—H82C	109.5
C13—C14—C15	121.0 (5)	H82A—C82—H82B	109.5
C16—C14—C13	117.5 (5)	H82A—C82—H82C	109.5
C16—C14—C15	121.5 (5)	H82B—C82—H82C	109.5
C14—C15—H15A	109.5	C81—C83—H83	118.9
C14—C15—H15B	109.5	C81—C83—C84	122.1 (5)
C14—C15—H15C	109.5	C84—C83—H83	118.9
H15A—C15—H15B	109.5	C77—C84—C85	120.8 (5)
H15A—C15—H15C	109.5	C83—C84—C77	117.1 (5)
H15B—C15—H15C	109.5	C83—C84—C85	122.1 (5)
C14—C16—H16	118.8	C84—C85—H85A	109.5
C17—C16—C14	122.4 (6)	C84—C85—H85B	109.5
C17—C16—H16	118.8	C84—C85—H85C	109.5
C16—C17—C18	120.9 (6)	H85A—C85—H85B	109.5
C16—C17—C19	118.6 (5)	H85A—C85—H85C	109.5
C19—C17—C18	120.4 (6)	H85B—C85—H85C	109.5
C17—C18—H18A	109.5	O1—C86—Ir3	170.7 (6)
C17—C18—H18B	109.5	C1—Ir1—H	93.9 (4)
C17—C18—H18C	109.5	C1—Ir1—HA	96.3 (4)
H18A—C18—H18B	109.5	C1—Ir1—HB	157.3 (13)
H18A—C18—H18C	109.5	C1—Ir1—HC	74 (2)
H18B—C18—H18C	109.5	C1—Ir1—HD	79 (2)
C17—C19—H19	119.1	C1—Ir1—Ir2	120.04 (13)
C17—C19—C20	121.8 (5)	C1—Ir1—Ir3	113.71 (14)
C20—C19—H19	119.1	C1—Ir1—Ir4	169.92 (14)
C13—C20—C19	117.3 (5)	C1—Ir1—Ir5	115.10 (14)
C13—C20—C21	122.5 (5)	H—Ir1—HA	66.7 (4)
C19—C20—C21	120.2 (5)	H—Ir1—HB	97 (4)
C20—C21—H21A	109.5	H—Ir1—HC	149.3 (19)
C20—C21—H21B	109.5	H—Ir1—HD	114.5 (18)
C20—C21—H21C	109.5	HA—Ir1—HB	106.3 (19)
H21A—C21—H21B	109.5	HA—Ir1—HC	86.1 (18)
H21A—C21—H21C	109.5	HA—Ir1—HD	175 (2)
H21B—C21—H21C	109.5	HB—Ir1—HC	104 (5)
N5—C22—Ir5	129.9 (4)	HB—Ir1—HD	78 (3)
N5—C22—N6	103.6 (4)	HC—Ir1—HD	91.5 (11)
N6—C22—Ir5	126.5 (4)	Ir2—Ir1—H	95.5 (3)
C24—C23—H23	126.7	Ir2—Ir1—HA	38.1 (3)
C24—C23—N6	106.6 (5)	Ir2—Ir1—HB	78 (4)
N6—C23—H23	126.7	Ir2—Ir1—HC	68 (2)
C23—C24—H24	125.9	Ir2—Ir1—HD	144 (2)
C23—C24—N5	108.1 (5)	Ir2—Ir1—Ir3	58.490 (7)
N5—C24—H24	125.9	Ir2—Ir1—Ir4	58.859 (7)
C26—C25—C33	122.9 (5)	Ir2—Ir1—Ir5	107.557 (8)
C26—C25—N5	117.2 (5)	Ir3—Ir1—H	37.2 (3)
C33—C25—N5	119.8 (5)	Ir3—Ir1—HA	37.3 (3)
C25—C26—C27	121.9 (6)	Ir3—Ir1—HB	86.7 (12)
C25—C26—C28B	119.9 (9)	Ir3—Ir1—HC	121.9 (18)
C28A—C26—C25	112.7 (12)	Ir3—Ir1—HD	145.9 (17)
C28A—C26—C27	124.2 (12)	Ir4—Ir1—H	76.5 (4)
C28B—C26—C27	116.8 (9)	Ir4—Ir1—HA	77.2 (4)
C26—C27—H27A	109.5	Ir4—Ir1—HB	30.9 (4)
C26—C27—H27B	109.5	Ir4—Ir1—HC	112 (2)
C26—C27—H27C	109.5	Ir4—Ir1—HD	107 (2)
H27A—C27—H27B	109.5	Ir4—Ir1—Ir3	56.578 (6)
H27A—C27—H27C	109.5	Ir5—Ir1—H	37.6 (3)
H27B—C27—H27C	109.5	Ir5—Ir1—HA	95.6 (3)
C26—C28A—H28A	118.6	Ir5—Ir1—HB	66 (5)
C26—C28A—C29A	122.8 (19)	Ir5—Ir1—HC	170 (2)
C29A—C28A—H28A	118.6	Ir5—Ir1—HD	87.4 (17)
C28A—C29A—C30	119.8 (17)	Ir5—Ir1—Ir3	58.513 (7)
C32A—C29A—C28A	121 (2)	Ir5—Ir1—Ir4	58.724 (7)
C32A—C29A—C30	119 (2)	C65—Ir2—Ir1	177.43 (15)
C26—C28B—H28B	120.4	C65—Ir2—HA	141.5 (4)
C29B—C28B—C26	119.2 (13)	C65—Ir2—HE	90.5 (6)
C29B—C28B—H28B	120.4	C65—Ir2—HF	90.2 (6)
C28B—C29B—C31	121.5 (13)	C65—Ir2—HG	93.6 (10)
C28B—C29B—C32B	118.1 (16)	C65—Ir2—Ir3	120.06 (15)
C32B—C29B—C31	120.1 (15)	C65—Ir2—Ir4	121.50 (15)
C29A—C30—H30A	109.5	Ir1—Ir2—HA	38.0 (3)
C29A—C30—H30B	109.5	Ir1—Ir2—HE	87.3 (6)
C29A—C30—H30C	109.5	Ir1—Ir2—HF	88.5 (6)
H30A—C30—H30B	109.5	Ir1—Ir2—HG	88.5 (10)
H30A—C30—H30C	109.5	HA—Ir2—HE	65.8 (11)
H30B—C30—H30C	109.5	HA—Ir2—HF	118.3 (9)
C29B—C31—H31A	109.5	HA—Ir2—HG	112.1 (5)
C29B—C31—H31B	109.5	HE—Ir2—HF	90.5 (7)
C29B—C31—H31C	109.5	HE—Ir2—HG	175 (2)
H31A—C31—H31B	109.5	HF—Ir2—HG	86 (2)
H31A—C31—H31C	109.5	Ir3—Ir2—Ir1	61.413 (7)
H31B—C31—H31C	109.5	Ir3—Ir2—HA	39.5 (3)
C29A—C32A—H32A	122.4	Ir3—Ir2—HE	93.9 (15)
C33—C32A—C29A	115 (3)	Ir3—Ir2—HF	149.3 (7)
C33—C32A—H32A	122.4	Ir3—Ir2—HG	86.7 (9)
C29B—C32B—H32B	117.3	Ir3—Ir2—Ir4	58.184 (7)
C29B—C32B—C33	125 (2)	Ir4—Ir2—Ir1	61.004 (7)
C33—C32B—H32B	117.3	Ir4—Ir2—HA	78.9 (4)
C25—C33—C32B	113.6 (11)	Ir4—Ir2—HE	144.4 (9)
C25—C33—C34	121.4 (5)	Ir4—Ir2—HF	103.3 (15)
C32A—C33—C25	123.3 (17)	Ir4—Ir2—HG	33.3 (3)
C32A—C33—C34	114.6 (16)	C86—Ir3—Ir1	120.4 (2)
C32B—C33—C34	124.2 (12)	C86—Ir3—H	100.4 (5)
C33—C34—H34A	109.5	C86—Ir3—HA	101.1 (4)
C33—C34—H34B	109.5	C86—Ir3—Ir2	120.7 (2)
C33—C34—H34C	109.5	C86—Ir3—HH	144.3 (4)
H34A—C34—H34B	109.5	C86—Ir3—HI	79.3 (5)
H34A—C34—H34C	109.5	C86—Ir3—HJ	80.1 (5)
H34B—C34—H34C	109.5	C86—Ir3—Ir4	177.8 (2)
C36—C35—C42	122.2 (5)	C86—Ir3—Ir5	118.4 (2)
C36—C35—N6	119.4 (4)	Ir1—Ir3—H	37.0 (3)
C42—C35—N6	118.4 (5)	Ir1—Ir3—HA	37.1 (3)
C35—C36—C37	120.8 (5)	Ir1—Ir3—HH	95.3 (4)
C35—C36—C38	117.8 (5)	Ir1—Ir3—HI	139.6 (6)
C38—C36—C37	121.4 (5)	Ir1—Ir3—HJ	139.6 (6)
C36—C37—H37A	109.5	H—Ir3—HA	66.3 (4)
C36—C37—H37B	109.5	H—Ir3—HH	109.1 (12)
C36—C37—H37C	109.5	H—Ir3—HI	109.9 (8)
H37A—C37—H37B	109.5	H—Ir3—HJ	176.0 (8)
H37A—C37—H37C	109.5	HA—Ir3—HH	108.8 (13)
H37B—C37—H37C	109.5	HA—Ir3—HI	176.2 (8)
C36—C38—H38	118.8	HA—Ir3—HJ	109.7 (8)
C39—C38—C36	122.3 (5)	Ir2—Ir3—Ir1	60.097 (7)
C39—C38—H38	118.8	Ir2—Ir3—H	96.9 (3)
C38—C39—C40	120.5 (5)	Ir2—Ir3—HA	39.4 (3)
C38—C39—C41	118.7 (5)	Ir2—Ir3—HH	75.8 (17)
C41—C39—C40	120.8 (5)	Ir2—Ir3—HI	143.4 (6)
C39—C40—H40A	109.5	Ir2—Ir3—HJ	79.5 (6)
C39—C40—H40B	109.5	Ir2—Ir3—Ir5	110.419 (8)
C39—C40—H40C	109.5	HH—Ir3—HI	72.2 (14)
H40A—C40—H40B	109.5	HH—Ir3—HJ	71.9 (13)
H40A—C40—H40C	109.5	HI—Ir3—HJ	74.1 (7)
H40B—C40—H40C	109.5	Ir4—Ir3—Ir1	61.481 (7)
C39—C41—H41	119.6	Ir4—Ir3—H	80.5 (4)
C42—C41—C39	120.8 (5)	Ir4—Ir3—HA	81.1 (4)
C42—C41—H41	119.6	Ir4—Ir3—Ir2	61.089 (7)
C35—C42—C41	118.2 (5)	Ir4—Ir3—HH	33.8 (4)
C35—C42—C43	121.0 (5)	Ir4—Ir3—HI	98.5 (4)
C41—C42—C43	120.8 (5)	Ir4—Ir3—HJ	99.2 (4)
C42—C43—H43A	109.5	Ir4—Ir3—Ir5	61.019 (7)
C42—C43—H43B	109.5	Ir5—Ir3—Ir1	60.353 (7)
C42—C43—H43C	109.5	Ir5—Ir3—H	39.0 (3)
H43A—C43—H43B	109.5	Ir5—Ir3—HA	97.3 (3)
H43A—C43—H43C	109.5	Ir5—Ir3—HH	77.2 (16)
H43B—C43—H43C	109.5	Ir5—Ir3—HI	79.3 (6)
N7—C44—Ir4	126.0 (4)	Ir5—Ir3—HJ	144.0 (7)
N7—C44—N8	103.1 (4)	C44—Ir4—Ir1	117.57 (14)
N8—C44—Ir4	130.0 (4)	C44—Ir4—HB	86.7 (6)
C46—C45—H45	125.9	C44—Ir4—Ir2	126.36 (14)
C46—C45—N8	108.2 (5)	C44—Ir4—HG	95.1 (11)
N8—C45—H45	125.9	C44—Ir4—Ir3	172.31 (14)
C45—C46—H46	126.5	C44—Ir4—HH	144.6 (6)
C45—C46—N7	107.1 (5)	C44—Ir4—HK	80.7 (13)
N7—C46—H46	126.5	C44—Ir4—HL	78 (3)
C48—C47—C54	121.8 (5)	C44—Ir4—Ir5	111.78 (14)
C48—C47—N7	119.3 (5)	Ir1—Ir4—HB	31.0 (4)
C54—C47—N7	118.6 (5)	Ir1—Ir4—HG	91.1 (11)
C47—C48—C49	121.0 (5)	Ir1—Ir4—HH	97.1 (4)
C47—C48—C50	118.2 (5)	Ir1—Ir4—HK	92.8 (11)
C50—C48—C49	120.8 (5)	Ir1—Ir4—HL	164 (3)
C48—C49—H49A	109.5	HB—Ir4—HG	97 (5)
C48—C49—H49B	109.5	HB—Ir4—HH	127.1 (16)
C48—C49—H49C	109.5	HB—Ir4—HK	85 (5)
H49A—C49—H49B	109.5	HB—Ir4—HL	165 (3)
H49A—C49—H49C	109.5	Ir2—Ir4—Ir1	60.137 (7)
H49B—C49—H49C	109.5	Ir2—Ir4—HB	80 (4)
C48—C50—H50	119.3	Ir2—Ir4—HG	37.3 (4)
C51—C50—C48	121.4 (5)	Ir2—Ir4—HH	76.2 (17)
C51—C50—H50	119.3	Ir2—Ir4—HK	147.5 (4)
C50—C51—C52	120.8 (6)	Ir2—Ir4—HL	108 (3)
C53—C51—C50	118.0 (5)	Ir2—Ir4—Ir5	110.040 (8)
C53—C51—C52	121.2 (5)	HG—Ir4—HH	91 (3)
C51—C52—H52A	109.5	HG—Ir4—HK	175.2 (6)
C51—C52—H52B	109.5	HG—Ir4—HL	83 (3)
C51—C52—H52C	109.5	Ir3—Ir4—Ir1	61.941 (7)
H52A—C52—H52B	109.5	Ir3—Ir4—HB	92.1 (12)
H52A—C52—H52C	109.5	Ir3—Ir4—Ir2	60.727 (7)
H52B—C52—H52C	109.5	Ir3—Ir4—HG	92.6 (11)
C51—C53—H53	118.4	Ir3—Ir4—HH	35.1 (4)
C51—C53—C54	123.2 (5)	Ir3—Ir4—HK	91.6 (12)
C54—C53—H53	118.4	Ir3—Ir4—HL	103 (3)
C47—C54—C55	121.5 (5)	Ir3—Ir4—Ir5	60.905 (7)
C53—C54—C47	117.4 (5)	HH—Ir4—HK	91 (3)
C53—C54—C55	121.1 (5)	HH—Ir4—HL	68 (3)
C54—C55—H55A	109.5	HK—Ir4—HL	94 (3)
C54—C55—H55B	109.5	Ir5—Ir4—Ir1	60.469 (7)
C54—C55—H55C	109.5	Ir5—Ir4—HB	67 (5)
H55A—C55—H55B	109.5	Ir5—Ir4—HG	147.3 (4)
H55A—C55—H55C	109.5	Ir5—Ir4—HH	77.8 (17)
H55B—C55—H55C	109.5	Ir5—Ir4—HK	37.5 (4)
C57—C56—N8	118.0 (5)	Ir5—Ir4—HL	120 (3)
C63—C56—C57	122.4 (5)	C22—Ir5—Ir1	174.45 (15)
C63—C56—N8	119.4 (5)	C22—Ir5—H	148.0 (4)
C56—C57—C58	120.7 (5)	C22—Ir5—Ir3	123.10 (14)
C59—C57—C56	118.2 (5)	C22—Ir5—Ir4	117.47 (14)
C59—C57—C58	121.1 (5)	C22—Ir5—HK	87.4 (9)
C57—C58—H58A	109.5	C22—Ir5—HM	89.6 (6)
C57—C58—H58B	109.5	C22—Ir5—HN	89.1 (6)
C57—C58—H58C	109.5	Ir1—Ir5—H	37.5 (3)
H58A—C58—H58B	109.5	Ir1—Ir5—HK	89.5 (10)
H58A—C58—H58C	109.5	Ir1—Ir5—HM	85.9 (6)
H58B—C58—H58C	109.5	Ir1—Ir5—HN	94.3 (6)
C57—C59—H59	119.0	H—Ir5—HK	111.4 (5)
C57—C59—C60	122.0 (6)	H—Ir5—HM	113.9 (8)
C60—C59—H59	119.0	H—Ir5—HN	70.3 (9)
C59—C60—C61	119.9 (6)	Ir3—Ir5—Ir1	61.135 (7)
C59—C60—C62	118.2 (6)	Ir3—Ir5—H	39.2 (3)
C62—C60—C61	122.0 (6)	Ir3—Ir5—Ir4	58.076 (7)
C60—C61—H61A	109.5	Ir3—Ir5—HK	85.4 (11)
C60—C61—H61B	109.5	Ir3—Ir5—HM	147.0 (6)
C60—C61—H61C	109.5	Ir3—Ir5—HN	93.7 (12)
H61A—C61—H61B	109.5	Ir4—Ir5—Ir1	60.807 (7)
H61A—C61—H61C	109.5	Ir4—Ir5—H	78.2 (4)
H61B—C61—H61C	109.5	Ir4—Ir5—HK	33.2 (3)
C60—C62—H62	119.0	Ir4—Ir5—HM	105.8 (14)
C60—C62—C63	122.0 (6)	Ir4—Ir5—HN	148.3 (9)
C63—C62—H62	119.0	HK—Ir5—HM	93 (2)
C56—C63—C62	117.2 (5)	HK—Ir5—HN	175 (2)
C56—C63—C64	121.4 (5)	HM—Ir5—HN	90.4 (7)
C62—C63—C64	121.4 (5)	C1—N1—C3	111.8 (4)
C63—C64—H64A	109.5	C1—N1—C4	129.4 (4)
C63—C64—H64B	109.5	C3—N1—C4	118.6 (4)
C63—C64—H64C	109.5	C1—N2—C2	111.7 (4)
H64A—C64—H64B	109.5	C1—N2—C13	128.7 (4)
H64A—C64—H64C	109.5	C2—N2—C13	119.0 (4)
H64B—C64—H64C	109.5	C65—N3—C77	125.3 (4)
N3—C65—Ir2	127.0 (4)	C66—N3—C65	112.0 (4)
N4—C65—Ir2	131.0 (4)	C66—N3—C77	122.6 (4)
N4—C65—N3	101.8 (4)	C65—N4—C67	111.7 (4)
C67—C66—H66	126.4	C65—N4—C68	128.2 (4)
C67—C66—N3	107.2 (5)	C67—N4—C68	120.1 (4)
N3—C66—H66	126.4	C22—N5—C24	110.7 (4)
C66—C67—H67	126.3	C22—N5—C25	125.9 (4)
C66—C67—N4	107.3 (5)	C24—N5—C25	122.4 (4)
N4—C67—H67	126.3	C22—N6—C35	127.4 (4)
C69—C68—N4	118.6 (4)	C23—N6—C22	111.0 (4)
C71—C68—C69	121.9 (5)	C23—N6—C35	121.1 (4)
C71—C68—N4	118.8 (5)	C44—N7—C46	111.3 (4)
C68—C69—C70	120.9 (5)	C44—N7—C47	129.0 (4)
C76—C69—C68	118.1 (5)	C46—N7—C47	118.7 (4)
C76—C69—C70	121.0 (5)	C44—N8—C45	110.3 (4)
C69—C70—H70A	109.5	C44—N8—C56	125.8 (4)
C69—C70—H70B	109.5	C45—N8—C56	122.2 (4)
C69—C70—H70C	109.5		
			
C2—C3—N1—C1	−0.8 (7)	C60—C62—C63—C64	177.5 (5)
C2—C3—N1—C4	176.0 (5)	C61—C60—C62—C63	179.4 (5)
C3—C2—N2—C1	0.6 (7)	C63—C56—C57—C58	−178.5 (5)
C3—C2—N2—C13	−171.0 (5)	C63—C56—C57—C59	1.3 (7)
C4—C5—C7—C8	2.5 (8)	C63—C56—N8—C44	−115.0 (5)
C5—C4—C11—C10	0.8 (8)	C63—C56—N8—C45	81.5 (6)
C5—C4—C11—C12	−177.6 (5)	C66—C67—N4—C65	0.6 (6)
C5—C4—N1—C1	−101.9 (6)	C66—C67—N4—C68	178.7 (5)
C5—C4—N1—C3	82.0 (6)	C67—C66—N3—C65	0.1 (6)
C5—C7—C8—C9	176.3 (5)	C67—C66—N3—C77	−176.3 (5)
C5—C7—C8—C10	−1.5 (8)	C68—C69—C76—C74	−2.1 (8)
C6—C5—C7—C8	−174.0 (5)	C68—C71—C73—C74	−1.0 (8)
C7—C8—C10—C11	0.2 (8)	C69—C68—C71—C72	−174.1 (5)
C8—C10—C11—C4	0.2 (8)	C69—C68—C71—C73	4.4 (8)
C8—C10—C11—C12	178.6 (5)	C69—C68—N4—C65	91.3 (6)
C9—C8—C10—C11	−177.6 (5)	C69—C68—N4—C67	−86.4 (6)
C11—C4—C5—C6	174.4 (5)	C70—C69—C76—C74	178.3 (5)
C11—C4—C5—C7	−2.1 (8)	C71—C68—C69—C70	176.7 (5)
C11—C4—N1—C1	87.5 (7)	C71—C68—C69—C76	−2.9 (8)
C11—C4—N1—C3	−88.6 (6)	C71—C68—N4—C65	−98.0 (6)
C13—C14—C16—C17	−2.5 (8)	C71—C68—N4—C67	84.3 (6)
C14—C13—C20—C19	5.6 (7)	C71—C73—C74—C75	173.8 (6)
C14—C13—C20—C21	−173.5 (5)	C71—C73—C74—C76	−3.8 (9)
C14—C13—N2—C1	−99.1 (6)	C72—C71—C73—C74	177.5 (6)
C14—C13—N2—C2	70.8 (6)	C73—C74—C76—C69	5.4 (9)
C14—C16—C17—C18	−172.8 (5)	C75—C74—C76—C69	−172.2 (6)
C14—C16—C17—C19	5.1 (8)	C77—C78—C80—C81	−1.5 (8)
C15—C14—C16—C17	176.3 (6)	C78—C77—C84—C83	−4.2 (8)
C16—C17—C19—C20	−2.4 (8)	C78—C77—C84—C85	177.0 (5)
C17—C19—C20—C13	−2.8 (8)	C78—C77—N3—C65	89.8 (6)
C17—C19—C20—C21	176.4 (5)	C78—C77—N3—C66	−94.3 (6)
C18—C17—C19—C20	175.5 (5)	C78—C80—C81—C82	175.8 (5)
C20—C13—C14—C15	178.1 (5)	C78—C80—C81—C83	−3.0 (8)
C20—C13—C14—C16	−3.1 (8)	C79—C78—C80—C81	178.6 (5)
C20—C13—N2—C1	87.8 (7)	C80—C81—C83—C84	4.1 (8)
C20—C13—N2—C2	−102.3 (6)	C81—C83—C84—C77	−0.6 (8)
C23—C24—N5—C22	−0.3 (7)	C81—C83—C84—C85	178.2 (5)
C23—C24—N5—C25	169.3 (5)	C82—C81—C83—C84	−174.8 (5)
C24—C23—N6—C22	−0.2 (7)	C84—C77—C78—C79	−174.9 (5)
C24—C23—N6—C35	−172.8 (5)	C84—C77—C78—C80	5.2 (8)
C25—C26—C28A—C29A	12.2 (18)	C84—C77—N3—C65	−91.2 (6)
C25—C26—C28B—C29B	−10.3 (15)	C84—C77—N3—C66	84.7 (6)
C26—C25—C33—C32A	12.9 (15)	Ir1—C1—N1—C3	−179.3 (4)
C26—C25—C33—C32B	−7.2 (12)	Ir1—C1—N1—C4	4.4 (8)
C26—C25—C33—C34	−177.2 (6)	Ir1—C1—N2—C2	179.3 (4)
C26—C25—N5—C22	88.5 (7)	Ir1—C1—N2—C13	−10.1 (8)
C26—C25—N5—C24	−79.4 (7)	Ir2—C65—N3—C66	175.0 (4)
C26—C28A—C29A—C30	175.3 (16)	Ir2—C65—N3—C77	−8.7 (7)
C26—C28A—C29A—C32A	−7 (3)	Ir2—C65—N4—C67	−175.0 (4)
C26—C28B—C29B—C31	−179.6 (12)	Ir2—C65—N4—C68	7.1 (8)
C26—C28B—C29B—C32B	6 (2)	Ir4—C44—N7—C46	168.4 (4)
C27—C26—C28A—C29A	179.8 (12)	Ir4—C44—N7—C47	−23.2 (7)
C27—C26—C28B—C29B	−176.9 (10)	Ir4—C44—N8—C45	−168.2 (4)
C28A—C29A—C32A—C33	3 (3)	Ir4—C44—N8—C56	26.7 (7)
C29A—C32A—C33—C25	−6 (3)	Ir5—C22—N5—C24	178.3 (4)
C29A—C32A—C33—C34	−176.7 (15)	Ir5—C22—N5—C25	9.2 (8)
C28B—C29B—C32B—C33	−3 (2)	Ir5—C22—N6—C23	−178.2 (4)
C29B—C32B—C33—C25	3 (2)	Ir5—C22—N6—C35	−6.2 (7)
C29B—C32B—C33—C34	172.6 (13)	N1—C1—N2—C2	−1.1 (6)
C30—C29A—C32A—C33	−179.1 (18)	N1—C1—N2—C13	169.5 (5)
C31—C29B—C32B—C33	−177.1 (14)	N1—C4—C5—C6	4.2 (7)
C33—C25—C26—C27	177.1 (6)	N1—C4—C5—C7	−172.3 (5)
C33—C25—C26—C28A	−15.0 (12)	N1—C4—C11—C10	171.0 (4)
C33—C25—C26—C28B	11.1 (11)	N1—C4—C11—C12	−7.4 (7)
C33—C25—N5—C22	−92.6 (7)	N2—C1—N1—C3	1.1 (6)
C33—C25—N5—C24	99.5 (6)	N2—C1—N1—C4	−175.2 (5)
C35—C36—C38—C39	0.1 (8)	N2—C2—C3—N1	0.1 (7)
C36—C35—C42—C41	−0.4 (8)	N2—C13—C14—C15	5.1 (8)
C36—C35—C42—C43	−178.8 (5)	N2—C13—C14—C16	−176.1 (5)
C36—C35—N6—C22	96.5 (6)	N2—C13—C20—C19	178.3 (4)
C36—C35—N6—C23	−92.2 (6)	N2—C13—C20—C21	−0.8 (7)
C36—C38—C39—C40	179.8 (5)	N3—C65—N4—C67	−0.5 (6)
C36—C38—C39—C41	0.2 (8)	N3—C65—N4—C68	−178.4 (5)
C37—C36—C38—C39	−176.4 (5)	N3—C66—C67—N4	−0.4 (6)
C38—C39—C41—C42	−0.7 (8)	N3—C77—C78—C79	4.0 (7)
C39—C41—C42—C35	0.8 (8)	N3—C77—C78—C80	−175.8 (5)
C39—C41—C42—C43	179.1 (5)	N3—C77—C84—C83	176.8 (4)
C40—C39—C41—C42	179.7 (5)	N3—C77—C84—C85	−2.0 (7)
C42—C35—C36—C37	176.5 (5)	N4—C65—N3—C66	0.3 (6)
C42—C35—C36—C38	0.0 (8)	N4—C65—N3—C77	176.5 (4)
C42—C35—N6—C22	−86.5 (7)	N4—C68—C69—C70	−12.9 (7)
C42—C35—N6—C23	84.9 (6)	N4—C68—C69—C76	167.6 (5)
C45—C46—N7—C44	1.3 (6)	N4—C68—C71—C72	15.5 (7)
C45—C46—N7—C47	−168.4 (5)	N4—C68—C71—C73	−166.0 (5)
C46—C45—N8—C44	−0.5 (6)	N5—C22—N6—C23	0.0 (6)
C46—C45—N8—C56	165.2 (5)	N5—C22—N6—C35	172.1 (5)
C47—C48—C50—C51	−0.4 (7)	N5—C25—C26—C27	−4.1 (9)
C48—C47—C54—C53	−0.3 (7)	N5—C25—C26—C28A	163.8 (9)
C48—C47—C54—C55	180.0 (5)	N5—C25—C26—C28B	−170.1 (8)
C48—C47—N7—C44	111.7 (6)	N5—C25—C33—C32A	−165.9 (14)
C48—C47—N7—C46	−80.6 (6)	N5—C25—C33—C32B	174.0 (10)
C48—C50—C51—C52	−178.2 (5)	N5—C25—C33—C34	4.0 (8)
C48—C50—C51—C53	1.1 (8)	N6—C22—N5—C24	0.2 (6)
C49—C48—C50—C51	179.9 (5)	N6—C22—N5—C25	−168.9 (5)
C50—C51—C53—C54	−1.4 (8)	N6—C23—C24—N5	0.3 (7)
C51—C53—C54—C47	1.0 (8)	N6—C35—C36—C37	−6.6 (8)
C51—C53—C54—C55	−179.3 (5)	N6—C35—C36—C38	176.9 (5)
C52—C51—C53—C54	177.9 (5)	N6—C35—C42—C41	−177.4 (5)
C54—C47—C48—C49	179.7 (5)	N6—C35—C42—C43	4.3 (7)
C54—C47—C48—C50	0.0 (7)	N7—C44—N8—C45	1.3 (5)
C54—C47—N7—C44	−74.7 (7)	N7—C44—N8—C56	−163.8 (5)
C54—C47—N7—C46	92.9 (6)	N7—C47—C48—C49	−7.0 (7)
C56—C57—C59—C60	−2.3 (8)	N7—C47—C48—C50	173.4 (4)
C57—C56—C63—C62	0.7 (7)	N7—C47—C54—C53	−173.7 (4)
C57—C56—C63—C64	−178.5 (5)	N7—C47—C54—C55	6.6 (7)
C57—C56—N8—C44	69.4 (6)	N8—C44—N7—C46	−1.5 (5)
C57—C56—N8—C45	−94.0 (6)	N8—C44—N7—C47	166.8 (5)
C57—C59—C60—C61	−177.3 (6)	N8—C45—C46—N7	−0.4 (6)
C57—C59—C60—C62	1.4 (8)	N8—C56—C57—C58	−3.1 (7)
C58—C57—C59—C60	177.5 (5)	N8—C56—C57—C59	176.7 (4)
C59—C60—C62—C63	0.7 (8)	N8—C56—C63—C62	−174.6 (4)
C60—C62—C63—C56	−1.7 (8)	N8—C56—C63—C64	6.1 (7)
